# Peptide-Based Vaccines for Tuberculosis

**DOI:** 10.3389/fimmu.2022.830497

**Published:** 2022-01-31

**Authors:** Wenping Gong, Chao Pan, Peng Cheng, Jie Wang, Guangyu Zhao, Xueqiong Wu

**Affiliations:** ^1^ Tuberculosis Prevention and Control Key Laboratory/Beijing Key Laboratory of New Techniques of Tuberculosis Diagnosis and Treatment, Senior Department of Tuberculosis, The 8th Medical Center of PLA General Hospital, Beijing, China; ^2^ State Key Laboratory of Pathogen and Biosecurity, Beijing Institute of Biotechnology, Beijing, China; ^3^ Hebei North University, Zhangjiakou City, China; ^4^ State Key Laboratory of Pathogen and Biosecurity, Beijing Institute of Microbiology and Epidemiology, Beijing, China

**Keywords:** tuberculosis (TB), peptide-based vaccine, epitope, bioinformatics, immunity, adjuvants, animal models

## Abstract

Tuberculosis (TB) is an infectious disease caused by *Mycobacterium tuberculosis*. As a result of the coronavirus disease 2019 (COVID-19) pandemic, the global TB mortality rate in 2020 is rising, making TB prevention and control more challenging. Vaccination has been considered the best approach to reduce the TB burden. Unfortunately, BCG, the only TB vaccine currently approved for use, offers some protection against childhood TB but is less effective in adults. Therefore, it is urgent to develop new TB vaccines that are more effective than BCG. Accumulating data indicated that peptides or epitopes play essential roles in bridging innate and adaptive immunity and triggering adaptive immunity. Furthermore, innovations in bioinformatics, immunoinformatics, synthetic technologies, new materials, and transgenic animal models have put wings on the research of peptide-based vaccines for TB. Hence, this review seeks to give an overview of current tools that can be used to design a peptide-based vaccine, the research status of peptide-based vaccines for TB, protein-based bacterial vaccine delivery systems, and animal models for the peptide-based vaccines. These explorations will provide approaches and strategies for developing safer and more effective peptide-based vaccines and contribute to achieving the WHO’s End TB Strategy.

## 1 Introduction

As an ancient infectious disease, tuberculosis (TB) has followed the footsteps of humanity for thousands of years (1, 2). *Mycobacterium tuberculosis* is the pathogen that causes TB. The war between humans and *M. tuberculosis* has never stopped from ancient times to modern society. Even today, TB remains a serious health threat. It has been reported that there were almost 1.3 million TB deaths among the human immunodeficiency virus (HIV) negative population in 2020 globally, up from 1.2 million in 2019 (3). These data indicated that the coronavirus disease 2019 (COVID-19) pandemic had disturbed years of global progress in reducing TB deaths, pushing the total number of TB deaths in 2020 back to the 2017 level (3). Furthermore, the emergence of drug-resistant and multidrug-resistant TB (MDR-TB) and the lack of effective methods for differential diagnosis of latent TB infection (LTBI) pose many challenges to TB prevention and treatment (4, 5).

Vaccination is the most effective way to control TB. The only licensed TB vaccine is Bacille Calmette-Guérin (BCG), which has been used for more than 100 years (6, 7). Previous studies have reported that BCG can protect children from severe TB and miliary TB. Therefore, BCG has been recommended by the World Health Organization (WHO) for widespread use in childhood immunization programmes in 154 countries in 2020 (3). However, a growing number of studies have reported that BCG is protective for only 10 - 20 years (8). This may be the reason why the defensive efficiency of BCG in adult pulmonary TB ranges from 0% to 80% (1, 9). According to the report released by WHO, there are 14 TB vaccine candidates in clinical trials, including AEC/BC02, Ad5 Ag85A, and ChAdOx185A-MVA85A vaccines in Phase I, MTBVAC, ID93 + GLA-SE, TB/FLU-04L, and GamTBvac vaccines in Phase IIa, DAR-901 booster, H56: IC31, M72/AS01_E_, BCG revaccination, and RUTI^®^ vaccines in Phase IIb, VPM1002 and MIP/Immuvac vaccines in Phase III (10). These TB novel vaccines can be divided into four categories: Viral vector vaccines, subunit vaccines, attenuated live mycobacterial vaccines, and inactivated vaccines (10). The most promising of these vaccines is M72/AS01_E_. It has been reported that the M72/AS01_E_ vaccine had good protection in healthy adults (11, 12), HIV infected adults in Switzerland (13), and healthy infants in Gambia (14). Consequently, M72 has been selected for further vaccine development. In 2018, a Phase IIb controlled trial of the M72/AS01_E_ vaccine showed that the protective efficacy of the vaccine against active pulmonary TB in adults was 54.0%, and there was no obvious safety problem (15). One year later, after three years of follow-up, the New England journal of medicine (NEJM) published the final results of this Phase IIb clinical trial of the M72/AS01_E_ vaccine. It was found that the total effectiveness was 49.7% after 36 months of follow-up, and the evaluation of vaccine efficacy increased throughout the study period, with vaccine effectiveness of 27.4% in the first year, 55.2% in the second year and 60.2% in the third year (16). However, it needs to be recognized that even if M72/AS01_E_ vaccine is proven to be reliable in larger populations, TB control cannot be based on the M72/AS01_E_ vaccine alone. We should develop more effective and safer vaccines to prevent and control TB.

**Figure 1:**
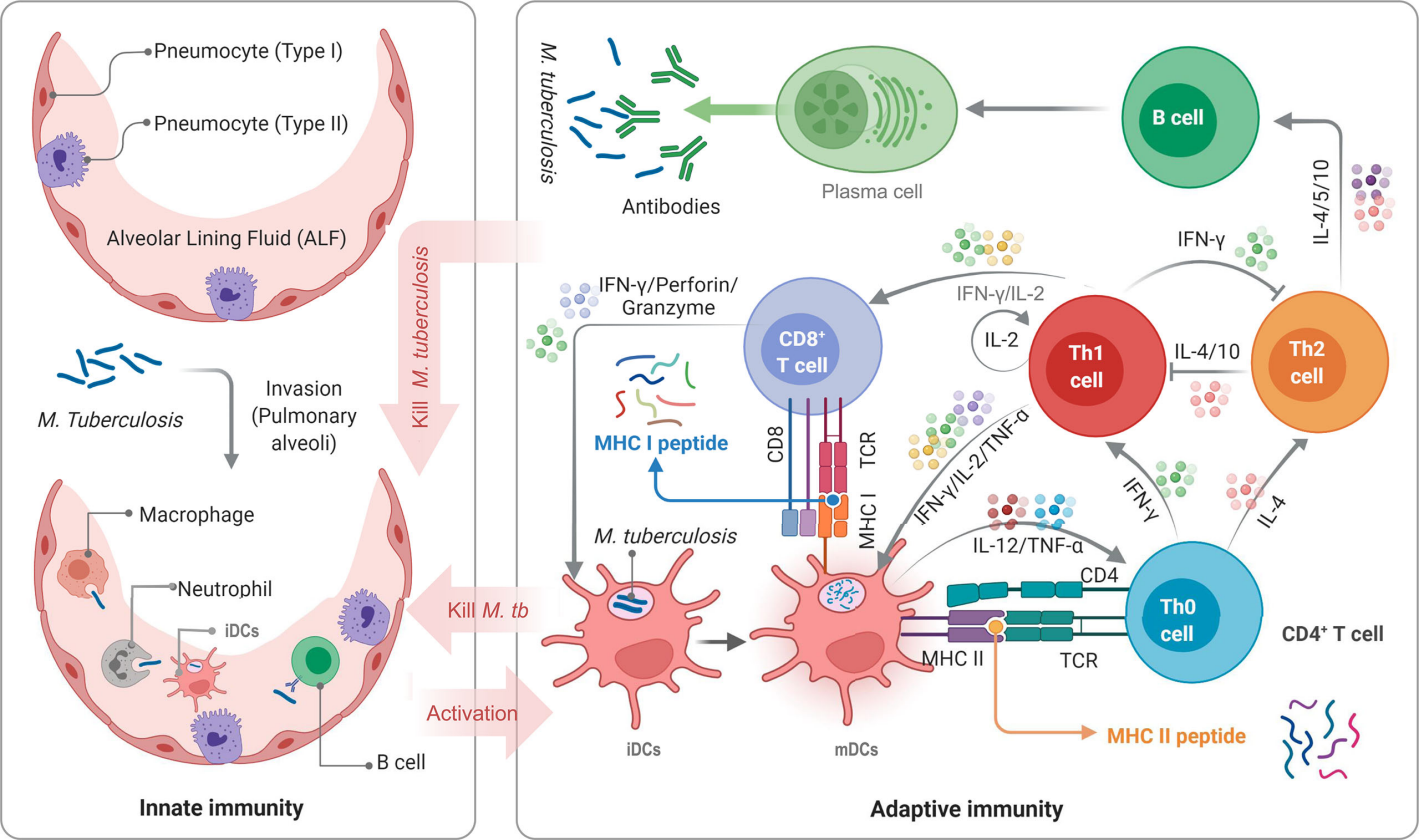
Schematic diagram of TB infection and anti-TB immunity of the host. APCs, such as DCs, macrophages, neutrophils, and even B cells, not only play the role of innate immune cells but also serve as a bridge between innate and adaptive immunity. DCs are the most important APCs, and their antigen presenting ability *in vitro* is 10-100 times that of other APCs. *M. tuberculosis* bacilli invading host’s pulmonary alveoli are first recognized and swallowed by APCs. Then, immature dendritic cells (iDCs) take up *M. tuberculosis* antigens and migrate to lymph nodes. During this process, the expression of MHC molecules on the surface is up-regulated, the antigen presentation function and the ability to activate T cells are also enhanced, and iDCs are transformed into mature dendritic cells (mDCs). mDCs can secrete interleukin-12 (IL-12), tumor necrosis factor-α (TNF-α), and interferon-α (IFN-α) to act on native CD4^+^ T cells (Th0 cells) to differentiate into Th1 cells. IFN-γ, IL-2, and TNF-α produced by Th1 cells can effectively activate CD8^+^ T cells and macrophages to eliminate intracellular *M. tuberculosis* by perforin, granzyme, reactive oxygen, and reactive nitrogen. Furthermore, mDCs produce IL-4, making Th0 cells differentiate into Th2 cells. The function of the Th2 response and lL-4 in the anti-tuberculosis immune response remains unclear. It is generally believed that Th2 cells will affect B lymphocytes by secreting IL-4, IL-5, and IL-10, mediating humoral immune response.

The biggest obstacle to developing a TB vaccine is the lack of understanding of the pathogenesis of *M. tuberculosis* and host immune protective mechanism. The innate and adaptive immunity of the host plays a vital role in the elimination or killing of *M. tuberculosis* (**Figure 1**). Innate immune cells, such as macrophages, dendritic cells (DCs), and natural killer (NK) cells, are the front-line to resist *M. tuberculosis* invasion. As the most important antigen presenting cells (APCs), macrophages and DCs play an essential role in phagocytosing *M. tuberculosis*. DCs activated by *M. tuberculosis* migrate to lymph nodes to display peptides of mycobacteria on their surface, which will be recognized by CD4^+^ T cells and CD8^+^ T cells through major histocompatibility complex (MHC) II and MHC I molecules, respectively (5, 17). Interestingly, the recognition between T cells and APCs is based on peptides rather than full-length protein (**Figure 1**). Therefore, the selection of vaccine candidate antigens and the prediction and screening of these immunodominant peptides are the key to designing a new generation of TB vaccine, known as peptide-based vaccine. The most significant advantage of peptide-based vaccines is the aggregation of immunodominant epitopes, which improves the immunogenicity of the vaccine and reduces its side effects (2).

This study reviewed the latest bioinformatics tools, linkers, and adjuvants used in designing peptide-based vaccines, the research status of peptide-based vaccines for TB, the protein-based bacterial vaccine delivery system, and animal models for peptide-based vaccines. To our knowledge, this is the first detailed and comprehensive review to report peptide-based vaccines for TB. We hope that through this review, we can provide practical tools and methods for designing peptide-based vaccines and contribute new ideas to developing novel TB vaccines.

## 2 The Development of Bioinformatics Technology Has Laid the Foundation for the Rise of Peptide-Based Vaccines for TB

The rapid development of bioinformatics technology makes it possible to predict and design a peptide-based vaccine with computers in advance. Compared with conditional vaccines, peptide-based vaccines have many advantages: more straightforward and faster production, more stable and convenient transportation and storage, lower cost, and decreased side effects (18). Despite these benefits of peptide-based vaccines, the following tasks must be done to design a peptide-based vaccine successfully: identification of potential antigens, prediction of T cell and B cell epitopes, analysis of epitope immunogenicity, antigenicity, allergenicity, and toxicity, selection of linkers and adjuvant peptides, construction and optimization of final vaccine, and analysis of the characteristics of the final vaccines. A list of databases or servers used to construct a peptide-based vaccine has been shown in **Table 1**.

**Table 1:** A list of databases or servers used to construct a peptide-based vaccine for TB.

Items	Subitems	Databases or servers	Web site	Remark	References
Protein sequence and functional information	Universal databases	NCBI	https://www.ncbi.nlm.nih.gov/protein	–	(19)
		Uniprot protein database	https://beta.uniprot.org/	–	(20, 21)
		GeneDB database	http://www.genedb.org/Homepage	–	(21, 22)
		Target-Pathogen database	http://target.sbg.qb.fcen.uba.ar/patho	Designed and developed as an online resource that allows the integration and weighting of protein information.	(23)
	Databases for *M. tuberculosis*	Mycobrowser database	https://mycobrowser.epfl.ch/	A comprehensive genomic and proteomic database for pathogenic mycobacteria	(20)
		MtbVeb	http://crdd.osdd.net/raghava/mtbveb/	A web portal for *M. tuberculosis* vaccines	(24)
		PeMtb	http://www.pemtb-amu.org	A practical platform for trial and computational analyses of antigenic peptides for *M. tuberculosis*	(25)
		MycobacRV	http://mycobacteriarv.igib.res.in	An immunoinformatics database of known and predicted mycobacterial vaccine candidates	(26)
MHC alleles	Population Coverage	Allele Frequency Net Database	http://www.allelefrequencies.net/pops.asp	–	(27)
		IEDB population coverage	http://tools.iedb.org/population/	–	(28)
T Cell epitope prediction tools	Epitope binding to MHC class II molecules (HTL epitope)	IEDB database	http://tools.immuneepitope.org/mhcii/	A small numbered adjusted percentile rank indicates high affinity, peptides with IC50 values <50 nM are considered high affinity	(29)
		RANKPEP server	http://imed.med.ucm.es/Tools/rankpep.HTML	Threshold 1.0: 49.5% sensitivity, 76.0% specificity; Threshold 0.5: 59.4% sensitivity, 69.4% specificity (Default); Threshold 0.0: 68.3% sensitivity, 60.9% specificity	(30)
		MetaMHCIIpan	http://datamining-iip.fudan.edu.cn/MetaMHCpan/index.php/pages/view/info	Peptides with IC50 less than 500 nm can be deemed as a binder.	(31)
		ProPred	http://www.imtech.res.in/raghava/propred/	The peptides predicted to bind > 50% HLA-DR alleles included in the ProPred were considered promiscuous for binding predictions.	(32, 33)
		NetMHCIIpan-4.0	https://services.healthtech.dtu.dk/service.php?NetMHCIIpan-4.0	The output of the model is a prediction score for the likelihood of a peptide to be naturally presented by and MHC II receptor of choice.	(34, 35)
		NetMHCIIpan 3.2	https://services.healthtech.dtu.dk/service.php?NetMHCIIpan-3.2	The prediction values are given in IC50 values and as %Rank, a lower % Rank value indicates a stronger binding peptide	(36)
	Epitope binding to MHC class I molecules (CTL epitope)	IEDB database	http://tools.immuneepitope.org/mhci/	A small numbered adjusted percentile rank indicates high affinity, peptides with IC50 values <50 nM are considered high affinity	(37)
		NetCTL-1.2	https://services.healthtech.dtu.dk/service.php?NetCTL-1.2	Different thresholds for the integrated score can be translated into sensitivity/specificity values.	(38)
		RANKPEP server	http://imed.med.ucm.es/Tools/rankpep.HTML	Threshold 1.0: 49.5% sensitivity, 76.0% specificity; Threshold 0.5: 59.4% sensitivity, 69.4% specificity (Default); Threshold 0.0: 68.3% sensitivity, 60.9% specificity	(30)
		ProPred1	http://www.imtech.res.in/raghava/propred1/	Mirror site of this server is available at http://bioinformatics.uams.edu/mirror/propred1/	(39)
		NetMHCpan-4.1	https://services.healthtech.dtu.dk/service.php?NetMHCpan-4.1	The peptide will be identified as a strong binder if it is found among the top x% predicted peptides, where x% is the specified threshold for strong binders (by default 0.5%).	(34)
		MetaMHCIpan	http://datamining-iip.fudan.edu.cn/MetaMHCpan/index.php/pages/view/info	Peptides with IC50 less than 500 nm can be deemed as a binder.	(31)
		NetMHC4.0	http://www.cbs.dtu.dk/services/NetMHC/	A default threshold value of 0.5% for strong binders and 2% for weak binders is recommended in NetMHC4.0	(40)
		MHCpred 2.0	http://www.ddg-pharmfac.net/mhcpred/MHCPred/	Suggested IC50 values are between 0.01 to 5000 nM. If the value is above 5000, then the peptide is unlikely to bind MHC molecules.	(41)
		EpiJen	http://www.ddg-pharmfac.net/epijen/EpiJen/EpiJen.htm	–	(42)
		CTLPred	http://www.imtech.res.in/raghava/ctlpred/index.html	A SVM and ANN based CTL epitope prediction	(43)
B Cell epitope prediction tools	Linear B cell epitopes	ABCpred	http://www.imtech.res.in/raghava/abcpred	Sensitivity = 67.14%, specificity = 64.71%, and accuracy = 66.41%.	(44, 45)
		IEDB Antibody Epitope Prediction	http://tools.iedb.org/bcell/	A collection of 7 methods to predict linear B cell epitopes based on sequence characteristics of the antigen using amino acid scales and HMMs.	(46–52)
		BCPred	http://ailab-projects1.ist.psu.edu:8080/bcpred/predict.html	AUC = 0.758, accuracy = 65.89%	(53, 54; a)
		BepiPred 2.0	https://services.healthtech.dtu.dk/service.php?BepiPred-2.0	AUC = 0.620	(52)
		APCpred	http:/ccb.bmi.ac.cn/APCpred/	AUC = 0.748 and accuracy = 68.43%	(55)
		SVMTriP	http://sysbio.unl.edu/SVMTriP/	Sensitivity = 80.1%, AUC = 0.702, and a precision of 55.2%	(56)
	Conformational B cell epitopes	DiscoTope-2.0	https://services.healthtech.dtu.dk/service.php?DiscoTope-2.0	AUC = 0.824 or 0.748 on the benchmark or Discotope dataset, respectively.	(57)
		BEpro (formerly known as PEPITO)	http://pepito.proteomics.ics.uci.edu/	AUC = 0.754 or 0.683 on the Discotope or Epitome dataset, respectively.	(58)
		ElliPro	http://tools.iedb.org/ellipro/	AUC = 0.732 on the benchmark dataset	(59)
		SEPPA	http://www.badd-cao.net/seppa3/index.html	AUC = 0.742 and a successful pick-up rate of 96.64%	(60)
		Epitopia	http://epitopia.tau.ac.il	AUC = 0.600	(61)
		EPCES	http://sysbio.unl.edu/EPCES/	Sensitivity = 47.8%, specificity = 69.5%, and AUC = 0.632.	(62)
		EPSVR	http://sysbio.unl.edu/EPSVR/	AUC = 0.597	(63)
		EPMeta	http://sysbio.unl.edu/EPMeta/	AUC = 0.638	(63)
Epitope Screening tools	Inducing MHC II binders’ prediction	IFNepitope	http://crdd.osdd.net/raghava/ifnepitope/index.php	Maximum prediction accuracy of 82.10% with MCC of 0.62 on main dataset	(64)
		IL4pred	https://webs.iiitd.edu.in/raghava/il4pred/index.php	Maximum accuracy of 75.76% and MCC of 0.51	(65)
		IL-10pred	https://webs.iiitd.edu.in/raghava/il10pred/predict3.php	MCC = 0.59 with an accuracy of 81.24%	(66)
	Immunogenicity	IEDB MHC I immunogenicity	http://tools.iedb.org/immunogenicity/	The higher score indicates a greater probability of eliciting an immune response	(67)
		IEDB MHC II immunogenicity	http://tools.iedb.org/CD4episcore/	predict the allele independent CD4 T cell immunogenicity at population level	(68)
		MARIA	https://maria.stanford.edu/	An integrated tool to predict how likely a peptide to be presented by HLA-II complexes on cell surface.	(69)
		PopCover-2.0	https://services.healthtech.dtu.dk/service.php?PopCover-2.0	An effective method for rational selection of peptide subsets with broad HLA and pathogen coverage	(70)
		BciPep	http://www.imtech.res.in/raghava/bcipep	A database of experimentally determined linear B-cell epitopes of varying immunogenicity	(71)
	Antigenicity	VaxiJen 2.0	http://www.ddg-pharmfac.net/vaxijen/VaxiJen/VaxiJen.html	The result will be showed as a statement of protective antigen or non-antigen	(72)
		ANTIGENpro	http://scratch.Proteomics.ics.uci.edu/	Correctly classifies 82% of the known protective antigens, accuracy on the combined dataset is estimated at 76%	(73)
	Allergenicity	AllerTOP v 2.0	http://www.ddg-pharmfac.net/AllerTOP/	The result will be showed as a statement of allergen or non-allergen	(74)
		AllergenFP v.1.0	http://ddg-pharmfac.net/AllergenFP/	The recognition accuracy was 88% and the Matthews correlation coefficient was 0.759.	(75)
		AlgPred 2.0	https://webs.iiitd.edu.in/raghava/algpred2/	The result will be showed as a statement of allergen or non-allergen	(76)
		Allermatch™	http://allermatch.org	The amino acid sequence of a protein of interest can be compared with sequences of allergenic proteins.	(77)
	Toxicity	ToxinPred	http://crdd.osdd.net/raghava/toxinpred/	The performance of dipeptide-based model in terms of accuracy was 94.50% with MCC 0.88	(78)
		T3DB	http://www.t3db.ca/biodb/search/target_bonds/sequence	Currently there are 3543 small molecule toxins (<1500 Da) and 136 peptide or protein toxins (>1500 Da) in T3DB	(79)
	Epitope Cluster Analysis	IEDB Clusters with Similar Sequences	http://tools.iedb.org/cluster/	This tool groups epitopes into clusters based on sequence identity	(80)
	Proinflammatory peptides	PIP-EL	http://www.thegleelab.org/PIP-EL/	MCC of 0.435 in a 5-fold cross-validation test	(81)
	Anti-inflammatory peptides	PreAIP	http://kurata14.bio.kyutech.ac.jp/PreAIP/	AUC = 0.833 in the training dataset *via* 10-fold cross-validation test, Score ≥ 0.468, Sensitivity = 63.22%; Specificity = 90.30%	(82)
Codon optimization and in silico cloning	Codon optimization	Java Codon Optimization Tool (JCat)	http://www.jcat.de/	The best CAI value is 1.0, while > 0.8 is regard a good score	(83)
	*In silico* clone	SnapGene software	https://www.snapgene.com/try-snapgene/		(84)
	Solubility prediction	Protein–Sol server	https://protein-sol.manchester.ac.uk/	Solubility value greater than 0.45 is predicted to have a higher solubility	(85)
Structure and function prediction	TCR-pMHC Binding Model	PAComplex	http://pacomplex.life.nctu.edu.tw./	Investigating and visualizing both TCR-peptide/peptide-MHC interfaces	(86)
		HADDOCK 2.2	http://haddock.science.uu.nl/services/HADDOCK/haddockserver-guru.html	Achieved success rate is 34.1%	(87)
		ClusPro server	https://cluspro.org	Achieved success rate is 27.3%	(88)
		LightDock	https://life.bsc.es/pid/lightdock/	Achieved success rate is 6.8%	(89)
		ZDOCK	http://zlab.bu.edu/~rong/dock	Achieved success rate is 15.9%	(90)
		iMOD	https://bio3d.colorado.edu/imod/paper/	NMA analysis of refined complexes	(91)
	Secondary structure prediction	PDBsum	http://www.ebi.ac.uk/thornton-srv/databases/pdbsum/		(92)
		SSpro8	http://scratch.proteomics.ics.uci.edu/	Can predict 8-class secondary structure of a protein	(93)
		GOR V server	https://abs.cit.nih.gov/gor/	Accuracy of prediction Q3 of 73.5%.	(94)
		SOPMA	http://npsa-pbil.ibcp.fr/cgi-bin/npsa_automat.pl?page=npsa_sopma.html		(95)
	Tertiary structure prediction	GalaxyWEB	http://galaxy.seoklab.org/cgi-bin/submit.cgi?type=REFINE	Protein structure prediction and refinement	(96)
		CABS-Flex 2.0	http://biocomp.chem.uw.edu.pl/CABSflex/	Predicts the structural flexibility of a protein/peptide	(97)
		3Dpro	http://scratch.proteomics.ics.uci.edu/	Predict tertiary structure of a protein	(98)
		Phyre2	http://www.sbg.bio.ic.ac.uk/phyre2	A typical structure prediction will be returned between 30 min and 2 h after submission	(99)
		SWISS-MODEL	http://swissmodel.expasy.org		(100)

AUC, area under the curve; CTL, cytotoxic T lymphocyte; HTL, helper T lymphocyte; IEDB, Immune Epitope Database and Analysis Resource; MCC, Matthews’ correlation coefficient; MHC, major histocompatibility complex; NCBI, National Center for Biotechnology Information; pMHCs, major histocompatibility complex presented antigenic peptides; PI, Protrusion Index.

### 2.1 Epitope Prediction

#### 2.1.1 Determination of Protective Antigens and the Coverage of MHC Alleles

Before predicting T cell or B cell epitopes, it is necessary to determine the protective antigens for vaccine construction and the coverage of MHC alleles in specific populations. Protective antigens should be selected from previous studies, and these antigens must have been proven to have significantly higher protective efficacy in animal models or clinical trials. Amino acid sequences of protective antigens can be obtained from National Center of Biotechnology Information (NCBI, https://www.ncbi.nlm.nih.gov/protein), Target-Pathogen database (http://target.sbg.qb.fcen.uba.ar/patho), Uniprot protein database (https://beta.uniprot.org/), and GeneDB database (http://www.genedb.org/Homepage) (19, 21–23).

Fortunately, with the rapid development of bioinformatics technology, a growing number of TB specific databases or servers have been developed for TB vaccine construction, such as Mycobrowser (20), MtbVeb (24), MycobacRV (26), and PeMtb (25). These novel tools provide powerful support for designing peptide-based vaccines for TB. Mycobrowser (https://mycobrowser.epfl.ch/) is a comprehensive genomic and proteomic database for ten pathogenic mycobacteria species, including *M. tuberculosis* H37Rv, *M. tuberculosis* 18b, *M. smegmatis* MC^2^-155, *M. orygis* 51145, *M. marinum* M, *M. lepromatosis* Mx1-22A, *M. leprae* TN, *M. haemophilum* DSM_44634/ATCC_29548, *M. bovis* AF2122/97, and *M. abscessus* ATCC_19977. The Mycobrowser knowledge base provides general annotation, gene or protein summary information, orthologues, and cross-references to the UniProt, Gene Ontology, SWISS-MODEL, and TB database (20). MtbVeb (http://crdd.osdd.net/raghava/mtbveb/) is a database developed by scientists from Institute of Microbial Technology in India for designing TB vaccines by using three approaches, such as strain, antigen, and epitope based vaccines (24). This database maintains integrated genomic information of 59 mycobacterium strains and provides comprehensive information for the antigenicity of potential vaccine candidates (24). MycobacRV database (http://mycobacteriarv.igib.res.in), developed by scientists from CSIR-Institute of Genomics and Integrative Biology in India, includes whole-genome sequences of 22 pathogenic mycobacterium species and one non-pathogenic mycobacterium H37Ra strain, and a set of 233 most probable vaccine candidates (26). Recently, a database of MHC antigenic peptide of *M. tuberculosis* named PeMtb has been developed to assist scientists in more efficient selection of epitopes that can be used for peptide-based vaccine construction (25). PeMtb is a free platform for predicting potential antigenic peptides of *M. tuberculosis*, which has unique advantages in epitopes prediction for TB vaccines development.

Furthermore, the most significant difference between peptide-based vaccines and traditional vaccines is that the former has MHC restriction. Human MHC molecules are also known as human leukocyte antigen (HLA). The HLA gene is located at the end of the short arm of human chromosome 6 and is divided into three regions: HLA Class I, HLA Class II, and HLA Class III (101). HLA genes with antigen presentation function are classic HLA genes located in HLA class I and HLA class II regions. The classic HLA I genes are divided into three categories: HLA-A, HLA-B, and HLA-C, and the classic HLA II genes are divided into three categories: HLA-DP, HLA-DQ, and HLA-DR (102). Therefore, selecting peptides with different MHC binding specificities will increase the coverage of the target population of peptide-based vaccines. However, due to the polymorphism of MHC molecules in other races, the design of peptide-based vaccines has become more complicated. Therefore, the coverage of MHC alleles in the vaccinated population must be considered when constructing a peptide-based vaccine. To overcome this issue, some databases and resources have been developed, including the Allele Frequency Net Database (http://www.allelefrequencies.net/pops.asp) and population coverage submodule of Immune Epitope Database and Analysis Resource (IEDB, www.iedb.org) (103). The Allele Frequency database provides allele frequencies for 115 countries and 21 different ethnicities grouped into 16 other geographical areas (28). IEDB is a popular database for providing information on immune epitopes. There are two components in the IEDB database, including the home page search and Analysis Resource. The home page search is designed to simplify the search process for many commonly queries such as Epitope (Linear peptide, discontinuous peptide, non-peptidic, and Any), Assay (T cell, B cell, and MHC ligand), Epitope Source (Organism and Antigen), MHC Restriction (Class I, Class II, Non-classical, and Any), hosts (humans, non-human primates, and other animal species), and Disease. The Analysis Resource component provides a set of tools for predicting and analyzing immune epitopes, which can be divided into three categories: (1) T Cell Epitope Prediction Tools: Peptide binding to MHC class I or II molecules (29, 37), peptide processing predictions and immunogenicity predictions (67, 104–106), TCRmatch (107), and structure tools such as LYRA (Lymphocyte Receptor Automated Modelling) (108), SCEptRe (Structural Complexes of Epitope Receptor) (109), and Docktope (109); (2) B Cell Epitope Prediction Tools: Prediction of linear epitopes from protein sequence including Chou & Fasman Beta-Turn Prediction, Emini Surface Accessibility Prediction, Karplus & Schulz Flexibility Prediction, Kolaskar & Tongaonkar Antigenicity, Parker Hydrophilicity Prediction, Bepipred Linear Epitope Prediction, and Bepipred Linear Epitope Prediction 2.0 (46–52); Discotope (110), ElliPro (59), methods for modeling and docking of antibody and protein 3D structures (111), LYRA server (108), and SCEptRe (109); (3) Analysis tools: Population Coverage (28), Epitope Conservancy Analysis (112), Epitope Cluster Analysis (80), Computational Methods for Mapping Mimotopes to Protein Antigens (http://tools.iedb.org/main/analysis-tools/mapping-mimotopes/), RATE (Restrictor Analysis Tool for Epitopes) (113), and ImmunomeBrowser (114). The components of the IEDB database related to peptide-based vaccine development are described in detail below.

#### 2.1.2 T Cell Epitope Prediction Tools

MHC molecules, expressed on the surface of APCs, are responsible for presenting antigenic peptides to T cells, making them irreplaceable in activating adaptive immunity (34). MHC molecules can be divided into two sets, MHC Class I (MHC I) and MHC Class II (MHC II), which primarily presents intracellular and extracellular peptides, respectively. Hence, identifying peptides binding to MHC I and II molecules is crucial for activating CD8^+^ and CD4^+^ T lymphocytes. Furthermore, recent studies have reported that engaging both helper T lymphocytes (HTL) epitopes binding to MHC II molecule and cytotoxic T lymphocytes (CTL) epitopes binding to MHC I molecule is desirable for inducing a robust immune response against *M. tuberculosis* (115, 116). Therefore, accurate computational prediction of HTL and CTL epitopes is the cornerstone for successfully constructing a peptide-based vaccine.

Currently, a growing number of bioinformatics technologies are available for HTL and CTL epitopes prediction, such as the IEDB database (29), RANKPEP server (30), MetaMHCIIpan (31), ProPred (32, 33), NetMHCIIpan-4.0 (34, 35), NetMHCIIpan 3.2 (36), NetCTL-1.2 (38), RANKPEP server (30), ProPred1 (39), NetMHCpan-4.1 (34), MetaMHCIpan (31), NetMHC4.0 (40), MHCpred 2.0 (41), EpiJen (42), and CTLPred (43) (**Table 1**). Three of these servers or databases can predict both HTL and CTL epitopes, including RANKPEP server, MetaMHCpan, and MHCPred. RANKPEP server predicts HTL and CTL epitopes from protein sequence using Position Specific Scoring Matrices (PSSMs) (30). MetaMHCpan has two parts: MetaMHCIpan and MetaMHCIIpan, for predicting CTL and HTL epitopes, respectively. MetaMHCIpan is based on two existing predictors (MHC2SKpan and LApan), while MetaMHCIIpan is based on four existing predictors (TEPITOPEpan, MHC2SKpan, LApan, and MHC2MIL) (31). MHCPred was developed to predict both HTL and CTL epitopes based on an Allele specific Quantitative Structure Activity Relationship (QSAR) model generated using partial least squares (PLS). MHCPred 2.0 covers 11 HLA class I, three human HLA class II, and three mouse MHC class I models (41). Furthermore, MHCPred 2.0 has multiple significant merits, such as incorporating a binding model for human transporter associated with antigen processing (TAP) that offers additional evidence, comprising a tool for designing heteroclitic peptides, and providing a confidence percentage for every peptide prediction (41).

As the most popular epitope prediction database, IEDB has unique advantages in HTL and CTL epitopes prediction. For MHC II epitopes prediction, nine methods are implemented, including IEDB recommended, Consensus method (117), Combinatorial library, NN-align-2.3 (netMHCII-2.3) (36), NN-align-2.2 (netMHCII-2.2) (118), SMM-align (netMHCII-1.1) (119), Sturniolo (120), NetMHCIIpan-3.1 (121), and NetMHCIIpan-3.2 (36). IEDB Recommended, selected as the default method, uses the best possible scenario for a given MHC molecule based on the following rules: the Consensus approach (a combination of any three of NN-align, SMM-align, CombLib, and Sturniolo) will be used if any corresponding predictor is available for the antigen. Otherwise, NetMHCIIpan is used. The performance of the MHC class II binding predictions has been evaluated in two studies based on over 10,000 binding affinities (117) and 40,000 binding affinities (29), and one study comparing pan-specific methods (122). For MHC I epitopes prediction, ten methods are implemented, including IEDB recommended 2020.09 (NetMHCpan EL4.1), Artificial neural network (ANN) (40), Stabilized matrix method (SMM) (123), SMM with a Peptide: MHC Binding Energy Covariance matrix (SMMPMBEC), Scoring Matrices derived from Combinatorial Peptide Libraries (Comblib_Sidney2008) (124), Consensus (125), NetMHCpan (126), NetMHCcons (127), PickPocket (128) and NetMHCstabpan (129). Similarly, IEDB recommended NetMHCpan EL 4.1 is selected as the default method and used across all alleles.

#### 2.1.3 B Cell Epitope Prediction Tools

More and more attention has been paid to the role of B cells in *M. tuberculosis* infection. B cells secrete antigen-specific antibodies to fight against *M. tuberculosis* invasion. Antigenic peptides are critical triggers for B cell antibody recognition. Therefore, the prediction of B cell epitopes is helpful to study the mechanism of the host’s self-protection system and design a peptide-based vaccine. Unlike T cell epitopes, B cell epitopes have both continuous (also known as linear epitopes) and discontinuous (also known as conceptual epitopes). A linear epitope is a continuous fragment from an antigen sequence. In contrast, a conformational epitope comprises several fragments distributed in an antigen sequence that form a structural domain-like interface in three dimensions.

At present, most of the available methods for predicting B cell epitopes are focused on continuous epitopes, such as ABCpred (44, 45), IEDB B-cell epitope tools (46–52), BCPred (53, 54; a), BepiPred 2.0 (52), APCpred (55), and SVMTriP (56). These methods are based on antigen amino acid sequence, and the operation method is simple and easy to study. Overall, the sensitivity and specificity of these methods for predicting linear B cell epitopes ranged from 60% to 70%, and the area under the curve (AUC) ranged from 0.6 to 0.8 (**Table 1**). Compared with other methods, IEDB Antibody Epitope Prediction is a collection of seven methods to predict continuous B cell epitopes based on antigen sequence using amino acid scales and machine learning algorithm Hidden Markov Model (HMM), including Bepipred Linear Epitope Prediction 2.0, Bepipred Linear Epitope Prediction, Chou & Fasman Beta-Turn Prediction, Emini Surface Accessibility Prediction, Karplus & Schulz Flexibility Prediction, Kolaskar & Tongaonkar Antigenicity, and Parker Hydrophilicity Prediction (46–52). These seven methods of IEDB predict linear B cell epitopes based on specific characteristics of an antigen sequence, such as hydrophilicity, accessibility, flexibility, turns, polarity, exposed surface, and antigenic propensity.

Previous studies have shown that up to 90% of B cell epitopes are discontinuous in nature, but most predictions have focused on linear epitopes, which may be related to the tertiary structure of proteins required for B cell conformational epitopes prediction. Despite the rapid development of single-crystal X-ray diffraction (SCXRD), nuclear magnetic resonance (NMR) spectroscopy, and X-ray crystallography, many tertiary structures of biological macromolecules have been elucidated, but accurate prediction of B cell epitopes remains challenging (58). Currently, several methods have been used for predicting conformational B cell epitopes, including DiscoTope-2.0 (57), BEpro (formerly known as PEPITO) (58), ElliPro (59), SEPPA (60), Epitopia (61), EPCES (62), EPSVR (63), EPMeta (63). A previous study compared the performance of DiscoTope-2.0 to the PEPITO, ElliPro, SEPPA, Epitopia, EPCES and EPSVR methods. The results indicated that the AUC value of DiscoTope-2.0 was observably higher than that of ElliPro but comparable to PEPITO. Furthermore, DiscoTope-2.0 revealed an enhanced AUC value compared to that of SEPPA (0.720 vs 0.711), EPCES (0.733 vs 0.695), Epitopia (0.727 vs 0.652) and EPSVR (0.746 vs 0.588) based on benchmark dataset (57).

#### 2.1.4 Peptide Analysis and Screening Tools

As shown in **Figure 1**, the IFN-γ and IL-4 cytokines secreted by APCs play an essential role in promoting the differentiation of native CD4^+^ T cells into Th1 and Th2 cells, which is the principal arm for controlling and killing intracellular *M. tuberculosis* (130). Three methods have been developed to predict the IFN-γ, IL-4, and IL-10 inducers by bioinformatics technologies, including IFNepitope (64), IL4pred (65), and IL-10pred (66). IFNepitope is an online prediction server that can predict the epitopes that can induce CD4^+^ T cells to secrete IFN-γ based on the protein sequence. It can help immunologists select and design IFN-γ-induced MHC Class II binding epitopes from proteins of interest, which is essential for designing better and more effective peptide-based vaccines (64). IL4pred and IL-10pred were developed to predict IL-4 and IL-10 inducing MHC II binding epitopes, respectively. The algorithm of the three servers relies on the following three models: Motif based model, Support Vector Machine (SVM) based model, and/or Hybrid approach (a combination of Motif and SVM). The maximum prediction accuracy of the three servers is 82.10%, 75.76%, and 81.24%, and the Matthew’s correlation coefficient (MCC) is 0.62, 0.51, and 0.59, respectively (64–66).

In the design of peptide-based vaccines, in addition to considering those mentioned cytokine-induced epitopes, it is also necessary to assess the immunogenicity, antigenicity, allergenicity, and toxicity of the epitopes. Previous studies have shown that these characteristics vary significantly among epitopes (67, 72, 74, 78). Therefore, how to choose epitopes with strong immunogenicity and antigenicity but low toxicity and allergenicity is a challenge for peptide-based vaccine design. To overcome these obstacles, some new algorithms, models or servers have been developed, including IEDB MHC I immunogenicity, IEDB CD4 T cell immunogenicity prediction, MARIA, BciPep, and PopCover-2.0 for immunogenicity (67–71), VaxiJen 2.0 and ANTIGENpro for antigenicity (72, 73), AllerTOP v 2.0, AllergenFP v.1.0, AlgPred 2.0, and Allermatch™ for allergenicity (74–77), ToxinPred and T3DB for toxicity (78, 79). In addition, other useful tools have been developed to help scientists design more effective peptide-based vaccines, such as IEDB Clusters with Similar Sequences for epitope cluster analysis (80), PIP-EL for proinflammatory peptides prediction (81), and PreAIP for anti-inflammatory peptides prediction (82).

### 2.2 Construction of Peptide-Based Vaccines

The most potential immunodominant epitopes are short peptides composed of dozens of amino acid residues and quickly degrade at the inoculation site. In order to overcome this shortcoming, it is necessary to use appropriate linkers and/or additional helper peptides (adjuvant peptides and agonists used in constructing a peptide-base vaccine) to fuse these dispersed epitopes to improve the efficiency of the vaccine.

#### 2.2.1 Linkers

Linkers are short amino acid sequences of natural origin that separate multiple domains in proteins (131). The selection of suitable linkers to link protein domains together is always complicated, but it is often overlooked in the design of peptide-based vaccines. If there are no linkers, a direct fusion of immunodominant epitopes may lead to many undesirable results, including misfolding of peptide-based vaccine (132), low vaccine yields (133), and impaired biological activity (134). Therefore, the selection and rational design of linkers connecting dominant epitopes is a crucial but undeveloped field in developing peptide-based vaccines.

According to the characteristics of linkers, they can be divided into three categories: flexible linkers, rigid linkers, and cleavable linkers. (1) Flexible linkers are usually used to connect protein domains that need mobility and interaction. They contain some polar or nonpolar amino acids with small molecular weight, which provides flexibility for the movement and interaction between protein domains (135). The commonly used flexible linkers include (GGGGS)_n_ (135), (Gly)_8_ (136), (Gly)_6_ (137), GSAGSAAGSGEF (138), KESGSVSSEQLAQFRSLD and EGKSSGSGSESKST (139). The GSAGSAAGSGEF linker is better than the (GGGGS)_4_ linker due to its better hydrophilicity and no-repeats (140). Providing flexibility for the movement and interaction between protein domains is the advantage of flexible linkers, but the lack of rigidity may lead to inefficient expression of recombinant proteins or loss of biological activity (133, 141). (2) Rigid linkers are usually used to maintain the distance between protein domains, effectively separating different protein domains and reducing the interaction and influence between the domains. Common rigid linkers are (EAAAK)n, A(EAAAK)_n_A (n = 2-5), PAPAP, (Ala-Pro)_n_ (132, 140, 142, 143). The rigid linker exhibits a relatively rigid structure by adopting an α-helical structure, and they separate protein domains more effectively than flexible linkers. Furthermore, the length of the rigid linker can be easily adjusted by changing the number of copies to achieve the best distance between domains. Therefore, rigid linkers are chosen when the spatial separation of domains is essential to maintain the stability or biological activity of the fusion protein. (3) Cleavage linkers are usually used to separate domains or peptides from protein or vaccine to achieve the biological functions of a single domain or peptide. These linkers can reduce steric hindrance, improve biological activity, and realize the independent function/metabolism of a single domain of the recombinant fusion protein after the linker is cut. However, the design of cleavable linkers for recombinant fusion proteins *in vivo* is a very challenging subject. Hence, cleavage linkers are rarely used in the design of peptide-based vaccines. Linkers used in peptide-based vaccine construction can be found in **Table 2**.

**Table 2:** Linkers and helper peptides used in peptide-based vaccine construction.

Reference	Helper peptides	Sequence of helper peptides	Linker for helper peptides	Linkers for epitopes			Diseases or pathogen
				CTL	HTL	B cell	
(144)	TLR2 agonist ESAT6	MTEQQWNFAGIEAAASAIQGNVTSIHSLLDEGKQSLTKLAAAWGGSGSEAYQGVQQKWDATATELNNALQNLARTISEAGQAMASTEGNVTGMFA	EAAAK	AAY	–	–	TB
(145)	TLR2 agonist PSMα4	MAIVGTIIKIIKAIIDIFAK	EAAAK	Alternately linked by GPGPG and AAY	–	–	TB
(115, 146)	TLR-2 agonist Pam2Cys	FNNFTVSFWLRVPKVSASHLE	NA	NA	NA	NA	TB
(147)	TLR2 agonist PorB	IALTLAALPVAAMADVTLYGTIKAGVETSRSVAHNGAQAASVETGTGIVDLGSKIGFKGQEDLGNGLKAIWQVEQ KASIAGTDSGWGNRQSFIGLKGGFGK	EAAAK	AAY	GPGPG	–	*Streptococcus pneumoniae*
(148)	TLR2 agonist PorB and helper epitope PADRE	PorB (IALTLAALPVAAMADVTLYGTIKAGVETSRSVAHNGAQAASVETGTGIVDLGSKIGFKGQEDLGNGLKAIWQVEQ KASIAGTDSGWGNRQSFIGLKGGFGK), and PADRE (AGLFQRHGEGTKATVGEPV)	EAAAK	GPGPG	AAY	–	*Neisseria meningitidis*
(149)	TLR4 agonist RpfE (Rv2450c)	LKNARTTLIAAAIAGTLVTTSPAGIANADDAGLDPNAAAGPDAVGFDPNLPPAPDAAPVDTPPAPEDAGFDPNLPPPLAPDFLSPPAEEAPPVPVAYSVNWDAIAQCESGGNWSINTGNGYYGGLRFTAGTWRANGGSGSAANASREEQIRVAENVLRSQGIRAWPVCGRRG	EAAAK	AAY	GPGPG	KK	TB
(150)	TLR4 agonist RplL	MAKLSTDELLDAFKEMTLLELSDFVKKFEETFEVTAAAPVAVAAAGAAPAGAAVEAAEEQSEFDVILEAAGDKKIGVIKVVREIVSGLGLKEAKDLVDGAPKPLLEKVAKEAADEAKAKLEAAGATVTVK	EAAAK	AAY	GPGPG	KK	TB
(151)	TLR4 agonist RplL and PADRE	RplL (MAKLSTDELLDAFKEMTLLELSDFVKKFEETFEVTAAAPVAVAAAGAAPAGAAVEAAEEQSEFDVILEAAGDKKIGVIKVVREIVSGLGLKEAKDLVDGAPKPLLEKVAKEAADEAKAKLEAAGATVTVK), PADRE (AGLFQRHGEGTKATVGEPV)	EAAAK	GPGPG	HEYGAEALERAG	–	*Schistosoma mansoni*
(152)	TLR4 agonist RplL	MAKLSTDELLDAFKEMTLLELSDFVKKFEETFEVTAAAPVAVAAAGAAPAGAAVEAAEEQSEFDVILEAAGDKKIGVIKVVREIVSGLGLKEAKDLVDGAPKPLLEKVAKEAADEAKAKLEAAGATVTVK	EAAAK	AAY	GPGPG	KK	*Staphylococcus aureus*
(153)	TLR4 agonist RplL	MAKLSTDELLDAFKEMTLLELSDFVKKFEETFEVTAAAPVAVAAAGAAPAGAAVEAAEEQSEFDVILEAAGDKKIGVIKVVREIVSGLGLKEAKDLVDGAPKPLLEKVAKEAADEAKAKLEAAGATVTVK	EAAAK	AAY	GPGPG	–	*Helicobacter pylori*
(154)	TLR4 agonist RplL	MAKLSTDELLDAFKEMTLLELSDFVKKFEETFEVTAAAPVAVAAAGAAPAGAAVEAAEEQSEFDVILEAAGDKKIGVIKVVREIVSGLGLKEAKDLVDGAPKPLLEKVAKEAADEAKAKLEAAGATVTVK	EAAAK	AAY	GPGPG	KK	*Leishmania parasite*
(155)	TLR4 agonist RplL	MAKLSTDELLDAFKEMTLLELSDFVKKFEETFEVTAAAPVAVAAAGAAPAGAAVEAAEEQSEFDVILEAAGDKKIGVIKVVREIVSGLGLKEAKDLVDGAPKPLLEKVAKEAADEAKAKLEAAGATVTVK	EAAAK	AAY	GPGPG	–	*Onchocerca volvulus*
(22)	TLR4 agonist RplL	MAKLSTDELLDAFKEMTLLELSDFVKKFEETFEVTAAAPVAVAAAGAAPAGAAVEAAEEQSEFDVILEAAGDKKIGVIKVVREIVSGLGLKEAKDLVDGAPKPLLEKVAKEAADEAKAKLEAAGATVTVK	EAAAK	GPGPG	AAY	–	*Schistosoma mansoni*
(156)	TLR4 agonist HBHA and helper epitope PADRE	HBHA (MAENSNIDDIKAPLLAALGAADLALATVNELITNLRERAEETRTDTRSRVEESRARLTKLQEDLPEQLTELREKFTAEELRKAAEGYLEAATSRYNELVERGEAALERLRSQQSFEEVSARAEGYVDQAVELTQEALGTVASQTRAVGERAAKLVGIEL), PADRE (AGLFQRHGEGTKATVGEPV)	EAAAK	GPGPG	GPGPG	–	Melanoma
(157)	TLR4 agonist CTB	MTPQNITDLCAEYHNTQIYTLNDKIFSYTESLAGKREMAIITFKNGAIFQVEVPGSQHIDQKKAIERMKDTLRIAYLTEAKVEKLCVWNNKTPHAIAAISMAN	EAAAK	GPGPG			Brucellosis
(158)	TLR4 agonist CTB	MTPQNITDLCAEYHNTQIYTLNDKIFSYTESLAGKREMAIITFKNGAIFQVEVPGSQHIDSQKKAIERMKDTLRIAYLTEAKVEKLCVWNNKTPHAIAAISMAN	EAAAK	–	GPGPG	–	*Vibrio cholerae*
(159)	TLR4 agonist CTB	MTPQNITDLCAEYHNTQIHTLNDKIFSYTESLAGKREMAIITFKNGATFQVEVPGSQHIDSQKKAIERMKDTLRIAYLTEAKVEKLCVWNNKTPHAIAAISMAN	GPGPG	GPGPG	GPGPG	KK	*Helicobacter pylori*
(160)	TLR-4 agonist RS-09	APPHALS	EAAAK	AAY	GPGPG	–	TB
(161)	Hsp70, TR-433, and TLR-4 agonist RS-09	Hsp70 (NTTIPTKRSETFTTADDNQPSVQIQVYQGEREIAAHNKFDIDANGIVHVTAKKDKGTGKENTAHAEEDRKRREEADVRNQAKFVKEQREAEGGSKV), TR-433 (NLKQMSEFSVFLSLRNLIYL), and RS-09 (APPHALS)	EAAAK	AAY	GPGPG	KK	SARS-CoV-2
(162)	HBD-1	MRTSYLLLFTLCLLLSEMASGGNFLTGLGHRSDHYNCVSSGGQCLYSACPIFTKIQGTCYRGKAKCCK	EAAAK	AAY	GPGPG	KK	SARS-CoV-2
(163)	HBD-2	MRVLYLLFSFLFIFLMPLPGVFGGIGDPVTCLKSGAICHPVFCPRRYKQIGTCGLPGTKCCKKP	EAAAK	GPGPG	–	–	HIV
(164)	HBD-3	GIINTLQKYYCRVRGGRCAVLSCLPKEEQIGKCSTRGRKCCRRKK	EAAAK	AAY	GPGPG	–	Zika virus
(165)	HBD-3	GIINTLQKYYCRVRGGRCAVLSCLPKEEQIGKCSTRGRKCCRRKK	EAAAK	AAY	GGGS	KK	Chandipura virus
(166)	HBD-3	GIINTLQKYYCRVRGGRCAVLSCLPKEEQIGKCSTRGRKCCRRKK	EAAAK	AAY	GPGPG	–	SARS-CoV-2
(167)	HBD-3	GIINTLQKYYCRVRGGRCAVLSCLPKEEQIGKCSTRGRKCCRRKK	EAAAK	GGGS	–	–	SARS-CoV-2
(19)	HBD-3	GIINTLQKYYCRVRGGRCAVLSCLPKEEQIGKCSTRGRKCCRRKK	EAAAK	AAY	GPGPG	KK	SARS-CoV-2
(84)	Griselimycin	VPSLPLVPLG	EAAAK	AAY	GPGPG	GPGPG	TB

CTB, Cholera toxin subunit B; HBHA, Heparin Binding Hemagglutinin; HDB, human β-defensin; PADRE, Pan human leukocyte antigen-DR reactive epitope; PSMα4, Phenol-soluble modulin α4; RplL, 50S ribosomal protein L7/L12; TLR: Toll-like receptor.

#### 2.2.2 TLR Agonists and Helper Peptides

**Figure 2:**
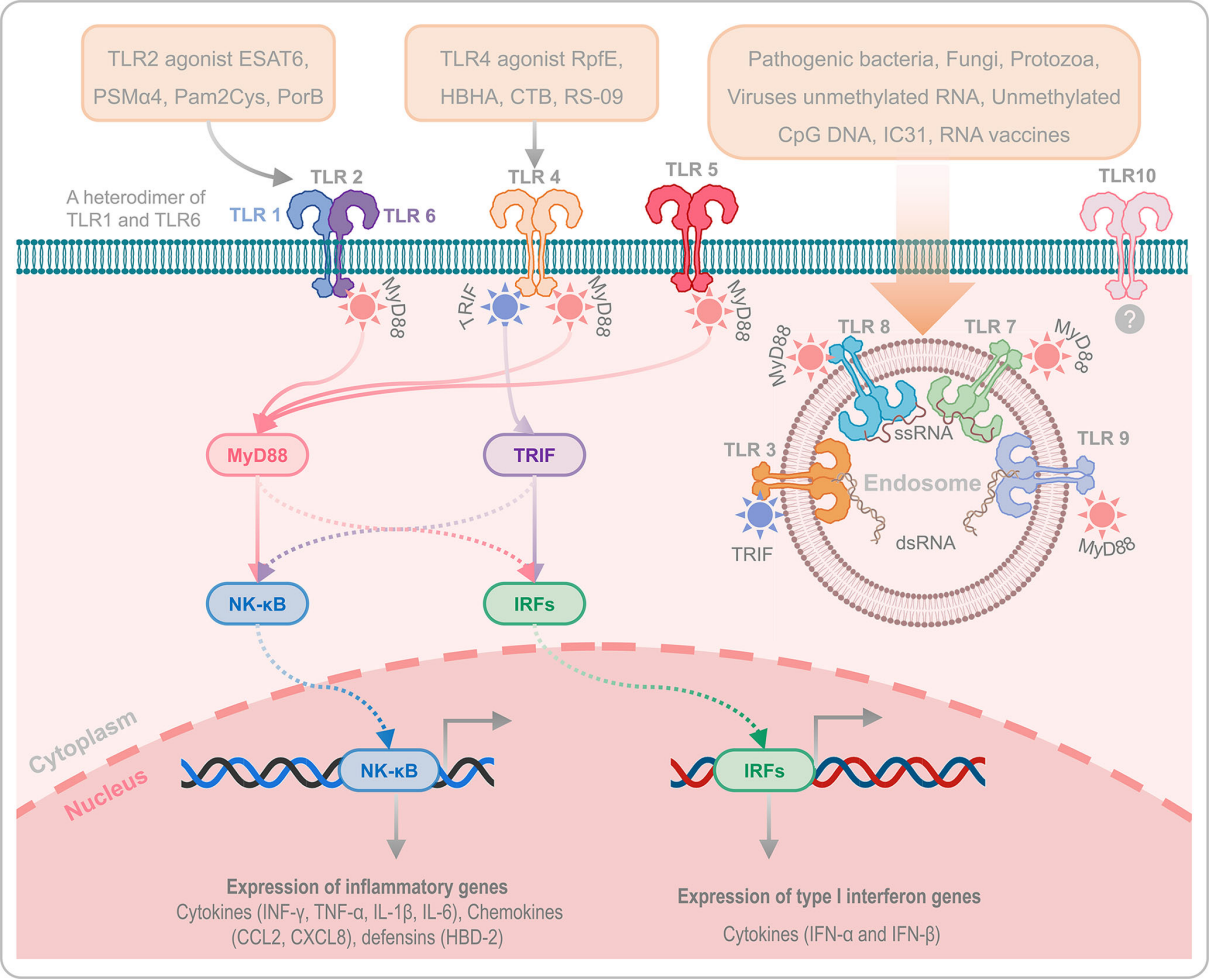
Toll-like receptors and their agonists. TLRs play an essential role in innate immunity and serve as a bridge between innate and adaptive immunity. TLR1, TLR2 (a heterodimer of TLR1 and TLR6), TLR4, TLR5, TLR6, and TLR10 are located on the cell membrane, while TLR3, TLR7, TLR8, and TLR9 are located on the membrane of the endosome. It has been reported that TLR2, TLR4, and TLR9 are critical for host defense against *M. tuberculosis* infection. In addition, the agonists of TLR2, TLR4, and TLR9 can enhance the immunogenicity and protective efficacy of peptide-based vaccines *via* TLR2- MyD88-NK-κB/IRFs-IFN-I/γ signaling pathway and TLR4-MyD88/TRIF-NK-κB/IRFs-IFN-I/γ signaling pathway. TLR10 is an orphan receptor without confirmed ligands, signalling pathway and biological function.

Like other subunit vaccines, weak immunogenicity is one of the disadvantages of peptide-based vaccines. Covalent conjugation of helper peptides to peptide-based vaccines appears to be a powerful strategy for improving the immunogenicity and protective efficiency of peptide-based vaccines. Currently, two kinds of adjuvants have been used in peptide-based vaccine construction to enhance its protective efficacy: Toll-like receptor (TLR) agonists and helper peptides. TLRs are important protein molecules involved in innate immunity and serve as a bridge between innate and adaptive immunity (168). There are ten common TLRs, of which, TLR1, TLR2 (a heterodimer of TLR1 and TLR6), TLR4, TLR5, TLR6 and TLR10 are located on the cell membrane, while TLR3, TLR7, TLR8, and TLR9 are located on the membrane of the endosome (**Figure 2**). Among these TLRs, TLR10 is an orphan receptor whose ligand, signaling pathway and biological function are still unknown (**Figure 2**).

Previous studies have reported that TLR2-, TLR4-, and TLR9-mediated immune responses are critical for host defense against *M. tuberculosis* infection (169, 170). TLR9, expressed by human B cells and DCs, play an essential role in recognizing the CpG DNA in bacterial rather than in not mammalian, which induces the differentiation, maturation, and proliferation of macrophages, NK cells, monocytes, T cells, and B cells, and enhances the production of Th1 type cytokines, such as IFN-γ, TNF-α, and IL-12 (171). More currently, there are three kinds of CpG oligonucleotides (ODN) that have been used as TLR-9 agonists, 1) Type A CpG ODN is consist of a phosphodiester/phosphothioate backbone, a palindrome sequence containing CpG dinucleotide, and poly G tail at the 3’ and 5’ terminals, which can activate the plasmacytoid dendritic cells (pDCs) to produce IFN-α (172); Type B CpG ODN is composed of multiple CpG motifs, which has solid immunostimulatory activity against B cells, but cannot activate pDCs (173); Type C CpG ODN is composed of whole phosphorothioate and palindromic CpG motifs, has the activity of both type A and type B CpG-ODNs, and can activate B cells and pDCs (174). TLR2 and TLR4 activate DCs by recognizing with the pathogen associated molecular patterns (PAMPs), and the activated DCs will produce kinds of cytokines to kill *M. tuberculosis via* TLR2- MyD88-NK-κB/IRFs-IFN-I/γ signaling pathway and TLR4-MyD88/TRIF-NK-κB/IRFs-IFN-I/γ signaling pathway (**Figure 2**) (175). Therefore, enhancing host immune responses with TLR2 and TLR4 agonists may be the option for constructing an ideal peptide-based vaccine in future. At present, some TLR2 and TLR4 agonists have been used in peptide-based vaccines against infectious diseases, including TB, such as TLR2 agonists ESAT6 (144), phenol-soluble modulin α4 (PSMα4) (145), dipalmitoyl-S-glyceryl cysteine (Pam2Cys) (115, 146), and PorB (147, 148), TLR4 agonists RpfE (Rv2450c) (149), 50S ribosomal protein L7/L12 (RplL) (22, 150–155), heparin binding hemagglutinin (HBHA) (156), cholera toxin subunit B (CTB) (157–159), and RS-09 (160, 161). In addition, helper peptides and antimicrobial peptides are also used to construct peptide-based vaccines to enhance their immune effects, such as PADRE (148) (151) (156), Hsp70 (161), TR-433 (161), human β-defensin 1 (HBD-1) (162), HBD-2 (163), HBD-3 (19, 164–167), and Griselimycin (84). The amino acid sequences of the mentioned helper peptides can be found in **Table 2**.

#### 2.2.3 Codon Optimization and Prediction of Structure and Function of Peptide-Based Vaccines

After the prediction and screening of dominant epitopes and the use of linkers and helper peptides (or adjuvants), a preliminary peptide-based vaccine was constructed. However, this native vaccine needs further optimization to become a mature peptide vaccine, including codon optimization, cloning and expression evaluation, and solubility prediction. Codon optimization is essential because the degeneracy of the genetic code allows one amino acid to be encoded by multiple codons (84). Java Codon Adaptation Tool (JCat) is the most popular tool for codon adaptation (**Table 1**). Compared with previous tools, JCat has superiorities in avoiding unnecessary cleavage sites for restriction endonuclease and Rho-independent transcription terminators and defining highly expressed genes as more intelligent, faster, and more accessible (83). Codon Adaptation Index (CAI) values are used to evaluate codon optimization. The best CAI value is 1.0, while CAI > 0.8 is regarded as a good score (83). Then, the sequence of the final vaccine optimized with JCat should be inserted into an appropriate plasmid vector using SnapGene software (84). Finally, the solubility of the final vaccine should be predicted by bioinformatics methods such as Protein-Sol server (85).

TCR and MHC are the bridges connecting APCs, T lymphocytes and peptide-based vaccines. Accurate recognition of TCR and major histocompatibility complex presented antigenic peptides (pMHC) triggers adoptive immune responses to kill *M. tuberculosis*. In the past, the crystallization and structural resolution of TCR-pMHC complexes were expensive and took a lot of time. However, with the development of computational technology, some valuable models or algorithms have been developed to study the TCR-pMHC interaction at the molecular level, such as PAComplex (86), ZDOCK (90), LightDock (89), ClusPro (88), HADDOCK (87) and iMOD (91). Furthermore, a recent study compared the ability of four standard tools (including ZDOCK, LightDock, ClusPro, and HADDOCK) to perform accurate molecular docking of the TCR-pMHC based on an expanded benchmark set of 44 TCR-pMHC docking cases (176). It was suggested that achieved success rates of HADDOCK, ClusPro, ZDOCK, and LightDock are 34.1%, 27.3%, 15.9% and 6.8%, respectively, indicating that HADDOCK is the best performer. At present, HADDOCK has been updated to version 2.2, which provides some new characteristics such as additional experimental restraints, support for mixed molecule types, improved protocols, and a more friendly interface (87).

The epitope prediction is based on the amino acid sequence of the protein. However, the immunological function of the peptide-based vaccine depends not only on the amino acid sequence but also on the secondary structure and tertiary structure of the vaccine candidate (177). Recently, some bioinformatics approaches and immunoinformatics technologies have been widely used in predicting the secondary structure and tertiary structure of peptide-based vaccines, including PDBsum (92), SSpro8 (93), GOR V server (94), and SOPMA (95) for secondary structure prediction, GalaxyWEB (96), CABS-Flex 2.0 (97), 3Dpro (98), Phyre2 (99), and SWISS-MODEL (100) for tertiary structure prediction.

## 3 Research Status of TB Peptide-Based Vaccines

Peptide-based vaccines are subunit vaccines and are new vaccines with unique advantages. Compared with traditional subunit vaccines, peptide-based vaccines are more precise and accurate in design (178). As mentioned above, the construction of peptide-based vaccines involves the identification of potential antigens, prediction and screening of dominant epitopes, comparison of MHC affinity, the addition of adjuvants or helper peptides, codon optimization, and prediction of structure and function. These tedious but indispensable processes enable peptide-based vaccines to efficiently cluster dominant epitopes together to induce a more robust immune response in the recipient, improving the efficiency and reducing side effects by excluding unwanted material from a full-length protein or whole pathogen (179).

**Figure 3:**
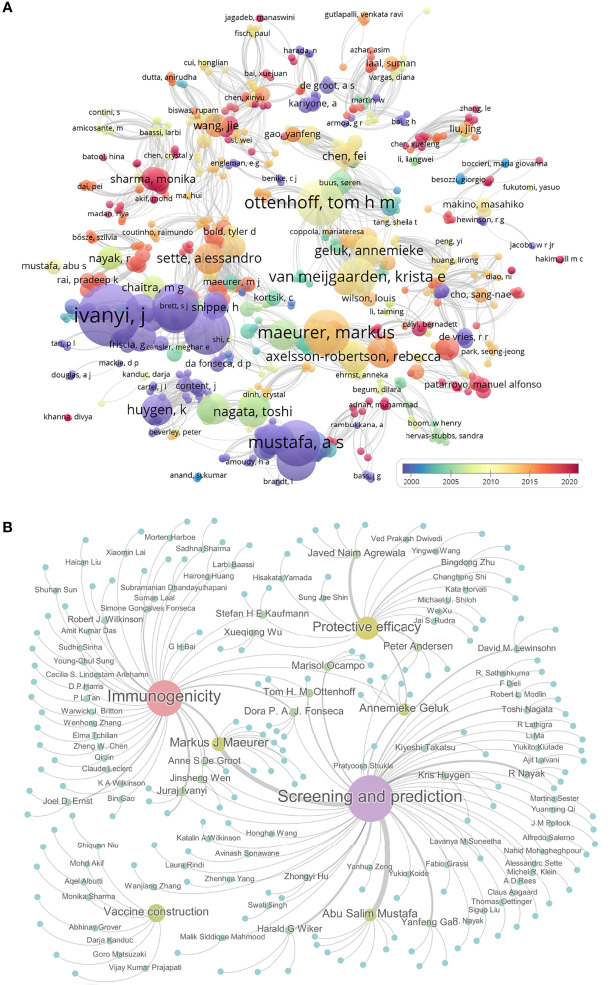
The progress in peptide-based vaccines for TB. The PubMed database was used to search the literature related to the peptide-based vaccine for TB using “peptide, epitope, and tuberculosis”. Their relationships were shown as bubble charts based on publication year and association strength by using VOSviewer software **(A)**. Furthermore, the relationships among 150 pieces of literature related to peptides or epitopes for TB were shown by using Gephi software. These literatures were divided into four categories: epitope screening and prediction, vaccine construction, immunogenicity, and protective efficacy. Gephi software was used to show 150 articles under corresponding authors. Each bubble represents a study, the size of which is proportional to the number of papers the author has published, and the color from blue to purple indicates the level of activity **(B)**.

The first peptide-based vaccine was reported and developed to fight against *Plasmodium falciparum* by Etlinger HM et al. in 1988 (180). This peptide-based vaccine consisted of a synthetic peptide [Ac-Cys-(NANP)3] and a tetanus toxoid protein. The immunological parameters of this vaccine were evaluated in a mouse model. To determine the research process of peptide-based vaccine for TB, we searched the PubMed database with terms of “peptide, epitope, and tuberculosis” (**Figure 3A**). Analyzing these results showed that the research of peptide-based vaccines for TB can be traced back to around 1990, but due to the lack of bioinformatics technology, the research progress is slow. Around 2010, with the rapid development of bioinformatics technology, the research of peptide-based vaccines for TB began to enter the fast lane. Especially in 2020, with the rise of COVID-19 peptide-based vaccines, the investigation of peptide-based vaccines for TB has also been extensively developed (red bubbles in **Figure 3A**).

Further analysis indicated that 150 studies involved in the peptide or epitope for TB, including 76 studies in epitope screening and prediction, 45 studies in evaluating immunogenicity, 8 studies in peptide-based vaccine construction, and 21 studies in assessing vaccine efficacy in animal models (**Figure 3B** and **Table S1**). Among these 150 articles, we found that 14 teams had published at least 3 articles in these four areas of TB epitopes or peptides, including six studies on epitope screening and prediction (181–186) and three studies on immunogenicity (187–189) from Pro. Markus J Maeurer’s team; seven studies on epitope screening and prediction from Pro. Abu Salim Mustafa’s team (190–196); one study on epitope screening and prediction (197) and two studies on immunogenicity (197, 198) from Pro. Anne S De Groot’s team; two studies on epitope screening and prediction (199, 200), one study on immunogenicity (201), and two studies on protective efficacy (202, 203) from Pro. Annemieke Geluk’s team; one study on epitope screening and prediction (204) and two studies on immunogenicity (205, 206) from Pro. Dora P. A. J. Fonseca’s team; three studies on epitope screening and prediction from Pro. Harald G Wiker’s team (207–209); four studies on protective efficacy from Pro. Javed Naim Agrewala’s team (115, 146, 210, 211); one study on epitope screening and prediction (212) and three studies on immunogenicity (212–214) from Pro. Juraj Ivanyi’s team; three studies on epitope screening and prediction from Pro. Kris Huygen’s team (215–217); two studies on epitope screening and prediction (218, 219) and one study on immunogenicity (220) from Pro. Marisol Ocampo’s team; one study on epitope screening and prediction (221) and two studies on protective efficacy (222, 223) from Pro. Peter Andersen’s team; four studies on epitope screening and prediction from Pro. R Nayak’s team (224–227); two studies on epitope screening and prediction (228, 229) and one study on protective efficacy (230) from Pro. Tom H. M. Ottenhoff’s team; three studies on epitope screening and prediction from Pro. Yanfeng Gao’s team (231–233). The works from these teams and the efforts of other scientists have laid the foundation for the development of peptide-based TB vaccines.

The detailed information of TB peptide-based vaccines in the stage of prediction, construction, and immunogenicity can be found in **Tables 2**, **S1**. Therefore, the following sections will focus on TB peptide-based vaccines in the stage of efficacy evaluation (**Table 3**).

**Table 3:** A list of TB peptide-based vaccines evaluated for immunogenicity and protection in animal models.

Reference	Protein and peptide (sequences)	Formulation (likers or adjuvant)	Host organism	Dose/route	Adjuvant	Challenge	Efficacy
(234)	Rv3407 _64-72_ (IPARRPQNL), and Rv3407 _68-76_ (RPQNLLDVT)	–	Splenocytes from vaccinated BALB/c mice	10^5^ splenocytes per well were stimulated with 10 μg of peptide	–	*-*	These peptides stimulated splenocytes collected from vaccine immunized mice secreting significantly higher IFN-γ
(197)	15 peptides^†^ from Rv0203, Rv3106, Rv2223c, Rv3201c, Rv3296, Rv1242, Rv1184c, Rv3207c, Rv1157c, Rv1158c, Rv1291c, Rv1860, Rv2190c, Rv333c, Rv0309	–	PBMCs	–	–	*-*	15 peptides stimulated IFN-γ response, and eight peptides stimulated lymphocyte proliferation *in vitro*.
(235)	pcDNA3-M-38 vaccine, MPT64 _190-198_ (FAVTNDGVI) and 38 kDa proteins _166-175_ (IAALNPGVNL)	pcDNA3 vector + MPT64 _190-198_ (FAVTNDGVI) + 38 kDa proteins _166-175_ (IAALNPGVNL)	C57BL/6 (H-2b) mice	100µg of DNA per mouse/i.m., 3 times at intervals of 21 days	–	–	DNA immunization with p3-M-38 vaccine could induce epitope-specific CD8^+^ CTL response but not antibodies
(236)	Ag85B _96–111_ (QDAYNAAGGHNAVFN) and Ag85B _241–256_ (PAFEWYYQSGLSIVMP)	Rv1886c _96-111_ or Rv1886c _241-256_ + RVG peptide (YTIWMPENPRPGTPCDIFTNSR)	C57BL/6 mice	10μg of peptides (RVG, Rv1886c _96-111_ or Rv1886c _241-256_)/s.c. or i.n., 3 times at 14days apart	–	–	Higher levels of IL-12, IFN-γ, IL-2, and TNF-α
(237)	Rv0934 _169-405_ and Rv0934 _802-1119_	Rv0934 _169-405_ + Rv0934 _802-1119_ + His-tag	BALB/c mice	Triplicate over a 2-week interval/s.c.	DDA/poly (I: C)	–	Elicited higher IgG and IFN-γ, IL-2
(198)	TB 001 DNA multi epitope vaccine, 24 peptides from Antigen 85 complex, MPT 64, MPB/MPT 70, MPT 63, the 38 kDa, 14-kDa, 16-kDa, 19-kDa, and 32-kDa Mtb	24 peptides linked with GPGPG linker	HLA-DR B*0101 transgenic mice	100 μg of DNA vaccine/i.m., 3 times at intervals of 14 days	rIL-15	*-*	Epitope-specific T cell responses were observed to eight of the 24 epitopes contained in the DNA construct
(223)	ESAT-6 (Rv3875) _1–15_ (MTEQQWNFAGIEAAA)	ESAT-6 or Δ15ESAT-6 (lack the immunodominant ESAT-6 _1–15_) + CAF01 adjuvant	CB6F1 mice	5 μg of ESAT-6 or Δ15-ESAT with a 200μl CAF01/s.c., 3 times, with a 2-week interval	CAF01	*Mtb* Erdman strain (20-50 CFUs/aerosol)	Both vaccines reduced CFUs at the early time point, only the Δ15ESAT-6-based vaccine gave significant levels of protection (0.9 log10 reduction of CFU)
(222)	ESAT-6 (Rv3875) _51–70_ (YQGVQQKWDATATELNNALQ)	DDA/MPLA/IL2 emulsion	B6CBAF1 mice	10μg peptide with a mixture of 25μg MPLA, and 100 ng recombinant mouse IL-2/i.p. or i.m., 3 times, with a 2-week interval	DDA and TLR4 agonist MPLA	*Mtb* H37Rv strain (5×10^4^ CFUs/i.v. or 250 CFUs/aerosol)	ESAT-6_51–70_ epitope promoted significant levels of protective immunity (equivalent to BCG and ESAT-6).
(238)	ESAT-6 (Rv3875) _4-18_ (QQWNFAGIEAAASAI), ESAT-6 _22-36_ (VTSIHSLLDEGKQSL) and ESAT-6 _56-70_ (QKWDATATELNNALQ)	pIRES + FL + ESAT-6 _4-18_ +AAY + ESAT-6 _22-36_ + AAY + ESAT-6 _56-70_ + HIS	C57BL/6 mice	100 µg plasmid DNA per mouse/i.m., two boosters at the interval of 3 weeks	–	*Mtb* H37Rv strain (5×10^5^ CFUs/intratracheal instillation)	DNA vaccine and boosted with the peptides increased IFN-γ and IL-12, the number of IFN-γ^+^ T cells and activities of CTL as well as IgG, enhanced protection challenge.
(239)	Ag85B (Rv1886c) _10-27_ (AWGRRLMIGTAAAVVLPG), Ag85B _19-36_ (TAAAVVLPGLVGLAGGAA), Ag85B _91-108_ (WDINTPAFEWYYQSGLSI), ESAT6 (Rv3875) _33-47_ (KQSLTKLAAAWGGSG), ESAT6 _37-51_ (TKLAAAWGGSGSEAY), ESAT6 _29-43_ (LDEGKQSLTKLAAAW), ESAT6 _72-95_ (LARTISEAGQAMASTEGNVTGMEA)	1 mL of vaccine mixture contains 10 μg of each peptide, 100 μg of Pam3Cys-SK-4, and 10 μg of CpG ODN	C57BL/6 mice	50 μl per mouse per dose	TLR9 agonist CpG ODN	*Mtb* H37Rv strain (150 CFUs/aerosol)	Enhanced BCG protective efficacy, induced Th1 and Th17 responses
(116)	Ag85B (Rv1886c) _239-247_ (KLVANNTRL), IniB (Rv0341) _33-45_ (GLIDIAPHQISSV) and PPE68 (Rv3873) _127-136_ (FFGINTIPIA)	Branched chain palmitoyl-peptide conjugate on Tuftsin (TKPKG) carrier, A/P/I mix, A(P)I, and Pal-A(P)I.	CB6F1 mice	50 μg vaccine in 100 μl PBS were injected s.c. three times, two weeks apart.	–	*Mtb* H37Rv strain (2×10^5^ CFUs/i.p.)	Significantly lower number of bacteria in the spleen after i.p. challenge with Mtb.
(240)	TB10.4 (Rv0288) _4–11_ (IMYNYPAM) and Ag85B (Rv1886c) _280–294_ (FQDAYNAAGGHNAVF)	TB10.4-KFE8 nanofibers or TB85B-KFE8 nanofibers with KFE8 (FKFEFKFE) + Pam2Cys adjuvant	C57BL6 mice	1 × 10^6^ CFU of BCG/s.c. prime followed by 25 μl of nanofiber formulations and boosted with 15 μl 30 or 90 days later	Pam2Cys	*Mtb* H37Rv strain (100 CFUs/aerosol)	Induced a 8-fold expansion in multifunctional CD8+ T cell populations and 1.3 log10 CFU reduction in lung bacterial burden.
(241)	Ag85A (Rv3804c) _141-160_	Recombinant (Ag85A) BCG Tokyo or Ag85A DNA vaccine with Ag85A peptide boosting	Guinea pigs	1. Recombinant (Ag85A) BCG: 5×10^6^ CFUs/s.c. boosted by 500 mg of Ag85A (141–160)/s.c. at 3 weeks later. 2. Ag85A DNA: 50 mg/i.m., 2 times at intervals of 3 weeks, boosted by 500 mg of Ag85A (141–160)/s.c. in IFA.	–	*Mtb* Kurono strain (150 CFUs/aerosol)	Peptide boosting is important for the induction of higher protective efficacy by recombinant BCG Tokyo or a tuberculosis DNA vaccine
(242)	Acr (Hsp16.3, Rv2031c) _91–104_ (SEFAYGSFVRTVSL)	Hsp16.3 _91–104_ peptide mixed with DDA-MPLA (TLR4 agonist) or IFA	BALB/c mice	25μg synthetic peptide with DDA-MPLA/mouse/s.c., 3 times, with a 2-week interval	250μg DDA + 25μg MPLA, or 100μl IFA	*Mtb* H37Rv strain (1×10^5^ CFUs/i.v.)	Induced significantly stronger specific antibodies but lower IFN-γ than BCG, the protection was equivalent to BCG
(210)	Acr (Rv2031c) _91–110_ (SEFAYGSFVRTVSLPVGADE)	Peptide + Pam2Cys	BALB/c mice or Duncan-Hartley guinea pigs	20 nmol per mouse or 100 nmol per guinea pig/i.p., 21 days later a booster (10 nmol per mouse and 50 nmol per guinea pig)	–	*Mtb* H37Rv strain (100 CFUs per mouse or 30 CFUs per guinea pigs, aerosol)	Enhanced activation of DCs, rousted Th1 immune response, and harbored significantly lower CFUs in the lungs
(146)	Acr (Rv2031c) _91–110_ (SEFAYGSFVRTVSLPVGADE)	L91 vaccine, 1 HTL (SEFAYGSFVRTVSLPVGADE) + TLR-2 agonist (Pam2Cys)	BALB/c mice	Danish strain of BCG (10^6^ CFU/animal), 21 days later, two boosters with L91 (20 nmol) at the interval of 2 weeks	–	*Mtb* H37Rv strain (100 CFUs/aerosol)	L91 booster significantly enhanced Th1 cells and Th17 cells and reduced the mycobacterial burden
(115)	Acr (Rv2031c) _91–110_ (SEFAYGSFVRTVSLPVGADE), TB10.4 (Rv0288) _20-28_ (GYAGTLQSL)	L4.8 vaccine, 1 HTL (SEFAYGSFVRTVSLPVGADE) + 1 CTL (GYAGTLQSL) + TLR-2 agonist (Pam2Cys)	BALB/c mice	Danish strain of BCG (10^6^ CFU/animal), 21 days later, two boosters with L4.8 (20 nmol) at the interval of 2 weeks	–	*Mtb* H37Rv strain (100 CFUs/aerosol)	Significantly elicited both CD8 T cells and CD4 T cells immunity, and the BCG-L4.8 prime boost strategy imparts a better protection against TB than the BCG alone.
(211)	TB10.4 (Rv0288) _1-13_ (MSQIMYNYPAMLG), TB10.4 (Rv0288) _78-94_ (ANTMAMMARDTAEAAKW), Rv0476 _1-19_ (MLVLLVAVLVTAVYAFVHA), CFP10 (Rv3874) _71-90_ (EISTNIRQAGVQYSRADEEQ), Acr (Rv2031c) _91-110_ (SEFAYGSFVRTVSLPVGADE), and Acr _21-40_ (LFAAFPSFAGLRPTFDTRLM)	All six peptide sequences aligned in duplicates were attached by protease-sensitive linker sequence with N terminal secretory signal of human growth hormone	C57BL/6 mice	100 μg per mouse/s.c. Two boosters at the interval of 2 weeks	–	*Mtb* H37Rv strain (100 CFUs/aerosol)	Significant reduction in the *Mtb* burden and enhanced IFN-γ and TNF-α cytokine release.
(202)	Hsp65 (Rv0440) _3–13_ (KTIAYDEEARR), Ag85B (Rv1886c) _56–64_ (PSMGRDIKV), 19 kDa (Rv3763) _51–61_ (KVVIDGKDQNV), Acr (Hsp16.3, Rv2031c) _31–50_ (LRPTFDTRLMRLEDEMKEGR) and Rv1733c _63–77_ (AGTAVQDSRSHVYAH)	Recombinant polyepitope with CpG ODN1826 adjuvant	HLA-DR3 transgenic mice	25 μg peptide vaccine with 50 μg CpG in 200 μl PBS were injected s.c. three times, two weeks apart.	50 μg TLR9 agonist CpG (ODN1826)	*Mtb* H37Rv strain (1×10^5^ CFUs/i.n.)	High IgG levels and polyfunctional CD4(+) T-cells producing IFN-γ, TNF and IL-2, and reduce CFUs in lungs
(243)	Four Th1 peptides ESAT-6 _1–20_ (MTEQQWNFAGIEAAASAIQG), Ag85B _241–255_ (VANNTRLWVYCGNGT), PE19 (Rv1791) _4–18_ (VTTQPEALAAAAANL), PPE25 (Rv1787) _241–255_ (AQFFASIAQQLTFGP), and 1 CTL peptide MTB10.4 (Rv0288) _3–11_ (QIMYNYPAM)	HSP65 scaffold + ESAT-6 _1–20_ + Ag85B _241–255_ + MTB10.4 _3–11_ + AAY + PPE25 _241–255_ + PE19 _4–18_	C57BL/6 mice	Four doses of 50 µg DNA vaccine per mouse/i.m.	–	Intranasally inoculated with 1 × 10^7^ CFUs BCG in 100 µL PBS under anesthesia	Induce higher IFN-γ^+^ T cell response, granzyme B^+^ CTL and IL-2^+^ CD8^+^ T cell responses, and significantly improved protection
(244)	21 conserved PE/PPE peptides ^‡^	PE peptide + ESAT-6 (PE-ESAT-6), PPE peptide + ESAT-6 (PPE-ESAT-6), and PE + PPE peptide + ESAT-6 (PE/PPE-ESAT-6)	C57BL/6J mice	2 mg per mouse/s.c. Two boosters at the interval of 3 weeks	GLA-SE (5mg/mouse)	*Mtb* Beijing strain HN878 (100 CFUs/aerosol)	Enhanced IL-2^+^IFN-γ^+^ CD4^+^ T cells, lower CFUs
(2)	Mtb8.4 (Rv1174c) _69-83_ (LRNFLAAPPPQRAAM), PPE18 _115-129_ (RAELMILIATNLLGQ), PPE18 (Rv1196) _149-163_ (AAAMFGYAAATATAT), PPE68 (Rv3873) _138-152_ (DYFIRMWNQAALAME), RpfA (Rv0867c) _377-391_ (AYTKKLWQAIRAQDV), and TB10.4 (Rv0288) _21-35_ (YAGTLQSLGAEIAVE)	TrxA-tag +6 HTL (GGGGS) + His-tag	Humanized C57BL/6 mice and wild- C57BL/6 mice	30 µg MP3RT per mouse/s.c. Two booster (20 µg) at the interval of 2 weeks	TLR9 agonist CpG-ODN2395	*Mtb* H37Rv strain (2 × 10^5^ CFUs/tail vein injection)	Inducing protection characterized by high levels of IFN-γ and CD3^+^IFN-γ^+^ T lymphocytes
(245)	MPT64 (Rv1980c) _190-198_ (FAVTNDGVI)	AMM (Ag85B-Mpt64 _190-198_ -Mtb8.4)	C57BL/6 mice	5 × 10^5^ CFU of BCG prime followed by 20 μg of AMM plus 250 μg of DDA and 30 μg of BCG PSN/s.c. Boosting twice at weeks 8 and 10	250 μg of DDA and 30 μg of BCG PSN	*Mtb* H37Rv strain (1×10^6^ CFUs/i.v.)	AMM induced stronger humoral and cell-mediated immune responses than Ag85B alone and could boost BCG-primed immunity and lead to a better protection than BCG alone or BCG-prime followed by Ag85B-boost.
(246)	MPT64 (Rv1980c) _190-198_ (FAVTNDGVI)	AMH (Ag85B-Mpt64_190-198_ -HspX) AMM (Ag85B-Mpt64 _190-198_ -Mtb8.4)	C57BL/6 mice	5 × 10^5^ CFU of BCG prime followed by 10 μg of AMM and 10 μg of AMH plus 250 μg of DDA and 30 μg of BCG PSN/s.c. Boosting twice at weeks 8 and 10	250 μg of DDA and 30 μg of BCG PSN	*Mtb* H37Rv strain (1×10^6^ CFUs/i.v.)	Boosted with AMM + AMH had significantly lower bacterial count in the lungs than those receiving BCG, whereas mice boosted with AMH or AMM did not.
(247)	MPT64 (Rv1980c) _190-198_ (FAVTNDGVI)	ESAT6 + Ag85B + MPT64_(190–198)_ + Mtb8.4-Rv2626c	C57BL/6 mice	13 μg/dose/mouse, s.c., 3 times at 2-week intervals	250 μg DDA and 50 μg TLR3 agonist Poly (I:C)	*Mtb* H37Rv strain (50-100 CFUs/aerosol)	Generated strong antigen-specific humoral and cell-mediated immunity, and induced higher protective efficacy than BCG
(203)	Rv1733c _57–84_ (IPFAAAAGTAVQDSRSHVYAHQAQTRHP)	Synthetic long peptide (SLP) with CpG ODN1826 adjuvant	HLA -DRB1*0301/DRA transgenic mice	25μg Rv1733c p63-77, or Rv1733c p57-84 peptide with CpG/mouse/s.c., 3 times, with a 2-week interval	TLR9 agonist CpG ODN1826	*Mtb* H37Rv strain (1×10^6^ CFUs/i.n.)	Had the highest reduction (0.92 log) in bacterial load in their lungs (from 3.6 × 10^5^ to 0.44 × 10^5^) compared to mice vaccinated only with BCG.

AcMNPV, Autographa californica multicapsid nucleopolyherovirus; BCG PSN, BCG polysaccharide nucleic acid; DDA, N, N’-dimethyl-N, N’-dioctadecylammonium bromide; FL, fms-like tyrosine kinase 3 ligand; IFA, Incomplete Freund’s Adjuvant; RVG, Rabies Virus Glycoprotein; MPLA, Monophosphoryl lipid A; Pam2Cys, lipid moiety S-[2,3-bis(palmitoyloxy)propyl] cysteine; PBMC, Peripheral blood mononuclear cells; p.i., postinfection; Poly (I: C), polyribocytidylic acid; s.c., subcutaneous injection; i.m., intramuscular injection; i.v., intravenous injection; i.n., intranasally; i.p., intraperitoneal injection; †: Rv0203 (TRRRLLAVLIAL), Rv3106 (GHRRMVFRFLTSPIEI), Rv2223c (WRRRPLSSALLSFGLLLGGLPL), Rv3201c (GQLLRRVRSRLARL), Rv3296 (RVILHSPYGLRVHGPLAL), Rv1242 (FLRIATSARVLAAPLPT), Rv1184c (LVPVNHLPLTLPL), Rv3207c (QGGLAPVMMQQTFST), Rv1157c (TQLLMAAASA), Rv1158c (GVNAPIPGI), Rv1291c (FTRRFAASMVG), Rv1860 (RKGRLAALAIA), Rv2190c (ARVIMRSAIG), Rv333c (VMRLYPVRLTTTMTR), Rv0309 (SVVMGVNKAK); ^‡^These 21 PE/PPE peptides can be found at https://doi.org/10.1016/j.bbrc.2018.06.017.

### 3.1 Peptide-Based TB Vaccines Evaluated for Immunogenicity

As early as 2004, Mollenkopf HJ et al. identified 36 DNA vaccine candidates preselected by comparative proteomic and found that BCG prime-Rv3407 encoding DNA vaccine boost vaccination induced significantly higher protection compared to BCG alone (234). Then, the putative MHC I epitopes of Rv3407 were predicted by computational method and determined by enzyme-linked immunospot assay (ELISPOT). It was found that Rv3407 _64-72_ (IPARRPQNL) and Rv3407 _68-76_ (RPQNLLDVT) peptides stimulated splenocytes collected from BALB/c mice immunized with Rv3407 DNA vaccine secreting significantly higher IFN-γ (234). Similarly, a year later, McMurry J et al. also identified 15 MHC II binding epitopes by bioinformatics tools and ELISPOT, these peptides from 15 antigens of *M. tuberculosis* could stimulate the PBMCs obtained from healthy or asymptomatic tuberculin skin test-positive donors producing high levels of IFN-γ (197). Although both studies simply validated potential peptides *in vitro*, they provide new insights into the development of peptide-based vaccines.

With the deepening of the understanding of epitopes, studies on evaluating the immunogenicity of peptides began to shift from *in vitro* experiments to *in vivo* experiments. Wang QM et al. constructed a pcDNA3-M-38 vaccine consisting of a pcDNA3 vector and two MHC II binding peptides MPT64 _190-198_ (FAVTNDGVI) and 38 kDa proteins _166-175_ (IAALNPGVNL). The results showed that immunization with the p3-M-38 vaccine could induce epitope-specific CD8^+^ CTL response in C57BL/6 (H-2b) mice (235). Recently, a study constructed a new peptide-based vaccine, PstS1p, which consists of PstS1 _169-405_ and PstS1 _802-1119_ peptides (237). The immunity and immunogenicity of the PstS1p vaccine and PstS1 (Rv0934) protein were evaluated in BALB/c mice. The results showed that both vaccines elicited remarkably higher levels of IgG antibodies and IFN-γ as well as IL-2 Th1-type cytokines (237). Interestingly, the PstS1p peptide-based vaccine showed more potent immunogenicity than the PstS1 vaccine, indicating that the peptide-based vaccine has better prospects than the traditional subunit vaccine.

These studies indicate an excellent method to construct peptide-based vaccines using bioinformatics technology to predict the dominant epitopes and ELSPOT technology for *in vitro* validation and screening. However, the immunogenicity of peptide-based vaccines containing only dominant epitopes is not ideal, and the addition of adjuvants or helper peptides can significantly improve the immunogenicity of peptide-based vaccines. Garnica O et al. used RVG peptide (YTIWMPENPRPGTPCDIFTNSR) to enhance the immunogenicity of Ag85B _96–111_ (QDAYNAAGGHNAVFN) and Ag85B _241–256_ (PAFEWYYQSGLSIVMP) peptides (236). They observed that stimulation with RVG peptide fused Ag85B _96–111_ or Ag85B _241–256_ peptide can improve the antigen presentation ability of mouse bone marrow derived DCs (BMDCs) and human THP-1 macrophages. Furthermore, the number of IFN-γ, IL-2, and TNF-α producing cells were significantly higher in mice immunized with RVG peptide fused Ag85B 241–256 peptide than in mice immunized with Ag85B _241–256_ peptide only (236). These results reveal that helper peptide RVG may be a promising adjuvant to developing effective peptide-based TB vaccines. The limitation of these studies is that the animal models used were wild-type mice. Still, the MHC molecules of mice and humans are significantly different, which may result in the failure of a peptide-based vaccine in clinical trials, which has been proved to have an excellent protective effect in the mouse model (2). To overcome this disadvantage, De Groot AS et al. developed HLA-DR B*0101 transgenic mice to evaluate the immunogenicity of a DNA multi-epitope vaccine that contains 24 epitopes derived from Antigen 85 complex, MPT 64, MPB/MPT 70, MPT 63, the 38 kDa, 14-kDa, 16-kDa, 19-kDa, and 32-kDa Mtb proteins (198). The results found that 8 of the 24 epitopes induced immune responses in these HLA-DR B*0101 transgenic mice.

### 3.2 Peptide-Based TB Vaccines Evaluated for Protective Efficacy in Animal Models

Previous studies on TB subunit vaccines have provided a large number of vaccine candidate antigens for peptide-based vaccines development, such as Ag85A (Rv3804c) (241), Ag85B (Rv1886c) (116, 202, 239, 240), 6-kDa early secretory antigenic target (ESAT-6, Rv3875) (222, 223, 238, 239), heat shock protein HspX (also known as Hsp16.3, Acr, and 14 kDa antigen, Rv2031c) (115, 146, 202, 210, 211, 242), TB10.4 (Rv0288) (2, 115, 211, 240), Rv0476 (211), Hsp65 (202), 19-kDa lipoprotein (Rv3763) (202), Rv1733c (202, 203), PE/PPE proteins (2, 243, 244, 248), MPT64 (Rv1980c) (246, 247), Mtb8.4 (2), and resuscitation-promoting factors (Rpfs) (2, 228). These antigens have been reported to be attractive vaccine candidates for preventing and controlling TB. Herein, we will summarize the peptide-based vaccines developed from these protective antigens.

#### 3.2.1 Peptide-Based Vaccines Derived From ESAT-6 Family Proteins

**E**SAT-6 family antigens are low-mass fractions of culture filtrates of *M. tuberculosis* (249). Previous studies reported that ESAT-6, CFP10, and TB10.4 antigens belong to ESAT-6 family members, they play an essential role in TB pathogenesis and induced significantly enhanced humoral and cellular responses in animal models or clinical trials (250–256). This evidence lay the foundation for constructing peptide-based vaccines based on **E**SAT-6 family antigens. Aagaard CS et al. identified an immunodominant peptide ESAT-6 (Rv3875) _1–15_ (MTEQQWNFAGIEAAA) that can be recognized by the splenocytes of CB6F1 mice and triggered a significant release of IFN-γ (223). To further investigate the role of ESAT-6 _1–15_ epitope in ESAT-6 full-length antigen, they compared the protective efficacy of a full-length ESAT-6 vaccine and a Δ15ESAT-6 vaccine with the absence of ESAT-6 _1–15_ epitope. Surprisingly, although both vaccines decreased bacterial numbers of the lung at the early time point, only the Δ15ESAT-6 vaccine revealed significant protection at the long time point (223). These data suggest that the ESAT-6 _1–15_ immunodominant epitope may negatively affect the full-length ESAT-6 vaccine, reminding that excluding some epitopes may be a potential approach to construct a more protective vaccine. Besides ESAT-6 _1–15_ epitope, other immunodominant epitopes of ESAT-6 antigen were also determined by different studies. Olsen AW et al. investigated the vaccine potential of two peptides, ESAT-6 _1–20_ (MTEQQWNFAGIEAAASAIQG) and ESAT-6 _51–70_ (YQGVQQKWDATATELNNALQ), in B6CBAF1 (H-2b,k) mice. The results showed that both peptides were recognized by CD4^+^ T cells and induced a significantly higher IFN-γ release, but only the vaccine based on the ESAT-6 _51–70_ peptide promoted significant protection against *M. tuberculosis* infection (222).

More recently, Jiang Q et al. constructed a recombinant DNA vaccine (pIRES-epitope-peptide-FL) encoding three T cell peptides of ESAT-6 antigen, including ESAT-6 _4-18_ (QQWNFAGIEAAASAI), ESAT-6 _22-36_ (VTSIHSLLDEGKQSL), and ESAT-6 _56-70_ (QKWDATATELNNALQ) peptides (238). Results revealed that pIRES-epitope-peptide-FL vaccination increased the proliferation of IFN-γ^+^ T cells, induced significantly higher levels of IFN-γ and IL-12 but relatively lower levels of IL-4 and IL-10, and enhanced protection from *M. tuberculosis* challenge in C57BL/6 mice (238). The results of the above studies suggest that there is competition between ESAT-6 _1–15_ or ESAT-6 _1–20_ epitope and other epitopes of ESAT-6 antigen, and ESAT-6 _1–15_ and ESAT-6 _1–20_ epitopes may weaken the recognition ability of other epitopes to MHC molecule (222). It may explain why the Δ15ESAT-6 vaccine or ESAT-6 51–70 peptide vaccine is more protective than the ESAT-6 vaccine or ESAT-6 _1–20_ peptide vaccine. These results provide a new strategy that synthetic long peptides (SLPs) in peptide-based vaccine design may attenuate the adverse effects of some epitopes and improve the immunogenicity and vaccine efficacy (116). Furthermore, ESAT-6 is a virulence factor secreted by *M. tuberculosis*, the safety of this protein and their peptides should be considered in designing a peptide-based vaccine.

#### 3.2.2 Peptide-Based Vaccines Derived From Ag85A and Ag85B Proteins

*M. tuberculosis* Ag85 complex consists of three homologous proteins, including Ag85A (Rv3804c), Ag85B (Rv1886c), and Ag85C (Rv0129c), which induce strong humoral and cellular immune responses (257). They play critical roles in virulence, preventing the formation of phagolysosomes and drug-resistant TB of the pathogen (258). Therefore, Ag85 complex proteins have been utilized by scientists to construct TB vaccines, such asrBCG30 (259), AERAS-422 (rBCG::Ag85A-Ag85B-Rv3407) (260), MVA85A (AERAS-485) (261), Ad35/AERAS-402 (262), Ad5Ag85A (262), ChAdOx1.85A (263), and AEC/BC02 (264). Previous studies have shown that Ag85A and Ag85B proteins are rich in epitope resources, giving them a distinct advantage in constructing peptide-based vaccines (257, 265). Kumar S et al. generated a vaccine mixture (peptide-TLR agonist-liposomes, abbreviation for PTL), consisting of three Ag85B peptides, four ESAT-6 peptides, TLR2 agonist Pam3Cys-SK-4, and TLR9 agonist CpG ODN (**Table 3**) (239). Interestingly, the BCG-PTL coimmunization enhanced the proportion of vaccine-induced Tcm cells and polyfunctional cytokine responses and increased the defensive efficiency against TB compared with BCG vaccination (239). Linear T cell epitopes are usually short and therefore less immunogenic and stable *in vivo*. It is an effective strategy to enhance the immunogenicity and presentation of epitopes by using conjugation and palmitoylation approaches. To investigate this strategy, Horváti K et al. developed three peptide-based vaccines termed A/P/I mix, A(P)I, and Pal-A(P)I, respectively (116). The similarity of the three vaccines is that they consist of Ag85B _239-247_ (KLVANNTRL), isoniazid inductible gene protein IniB (Rv0341) _33-45_ (GLIDIAPHQISSV), and PPE68 (Rv3873) _127-136_ (FFGINTIPIA) peptides. The difference is that A/P/I mix is a mixture of three peptides, A(P)I is a conjugation of three peptides separated with a tuftsin sequence (TKPKG), and Pal-A(P)I is the palmitoylated A(P)I. As expected, the internalization rates of A(P)I and Pal-A(P)I vaccines were higher than these of A/P/I mix in human murine bone marrow-derived macrophages (BMDMs) or MonoMac6 human monocytes (MM6), especially the Pal-A(P)I vaccine. Immunization with Pal-A(P)I vaccine induced significantly higher levels of splenocytes proliferation and Th1-type cytokines, and lower numbers of bacteria in the lung or spleen of mice. This exploration suggests that conjugation and palmitoylation are a promising route to enhance the immunogenicity and protective efficacy of peptide-based vaccine.

In addition to the conjugation and palmitoylation routes described above, other novel vaccination strategies have been developed to design peptide-based vaccines. Recently, Chesson CB et al. reported a vaccination strategy based on self-assembling peptide-nanofibers to present TB10.4 (Rv0288) _4–11_ (IMYNYPAM) and Ag85B (Rv1886c) _280–294_ (FQDAYNAAGGHNAVF) peptides (240). It was found that intranasal immunization with self-assembling peptide-nanofibers induced an 8-fold expansion in multifunctional CD8^+^ T cell populations and bacterial loads in the lungs of mice primed with BCG and boosted intranasally with co-assembled nanofibers of TB10.4 _4–11_ peptide and Pam2Cys adjuvant showed a 1.3 log10 CFUs reduction compared to naïve mice (240). Thus, the utilization of new materials or adjuvants can significantly improve the immunogenicity and protection efficiency of peptide-based vaccines. Furthermore, the BCG prime-boost strategy can also considerably enhance the protection efficiency of peptide-based vaccines. Sugawara I et al. confirmed the prime-boost strategy by developing a recombinant (Ag85A) BCG vaccine, an Ag85A DNA vaccine, and an Ag85A (Rv3804c) _141-160_ peptide (241). The results presented that the recombinant BCG Tokyo (Ag85A) or Ag85A DNA vaccination boosted with Ag85A _141-160_ peptide could remarkably reduce pathological lesions and CFUs burden in the lung of guinea pigs.

#### 3.2.3 Peptide-Based Vaccines Derived From 16-kDa Alpha-Crystallin (Acr, Rv2031c) Protein

Acr, also known as HspX and Hsp16.3, is a heat shock protein localized in the inner membrane. This protein plays an essential role in maintaining the long-term survival during latent or asymptomatic infection and slowing the growth of *M. tuberculosis* in the early stage of active disease (266, 267). Previous study has suggested it as a subunit vaccine candidate (268). Currently, three peptides from Acr protein have been used to develop peptide-based vaccines, including Acr _31–50_ (LRPTFDTRLMRLEDEMKEGR), Acr _91–104_ (SEFAYGSFVRTVSL), and Acr _91–110_ (SEFAYGSFVRTVSLPVGADE) peptides (**Table 3**). Shi C et al. compared the immune responses and protection of Acr protein vaccine and its peptide Acr _91–104_ mixed with TLR4 agonist DDA-MPLA (N, N’-dimethyl-N, N’-dioctadecylammonium bromide-Monophosphoryl lipid A) on BALB/c mice. They observed that, compared to the BCG vaccine, both vaccines induced significantly higher levels of antibodies, splenolymphocyte proliferation, lower levels of IFN-γ, and equivalent protection (242). Two previous studies constructed a peptide-based vaccine termed L91 by linking Acr _91–110_ (SEFAYGSFVRTVSLPVGADE) peptide to TLR-2 agonist Pam2Cys. Both studies found similar results that L91 vaccination stimulated significantly higher levels of Th1 and Th17 immune responses and induced significantly lower CFUs in the lungs of BALB/c mice or Duncan-Hartley guinea pigs than BCG vaccine (146, 210). The possible immune protective mechanism of the L91 vaccine is to reduce the inhibitory effect of *M. tuberculosis* on APCs by enhancing the expression of NF-κB and iNOS (146, 269). Interestingly, to achieve better protection, Rai, PK et al. improved the L91 vaccine by incorporating a CD8 T cell epitope TB10.4 _20-28_ (GYAGTLQSL), and the new peptide-based vaccine was named as L4.8 (115). The results showed that the L4.8 vaccination elicited significantly higher levels of CD4^+^ and CD8^+^ T cells immunity, and the BCG-L4.8 prime-boost strategy resulted in better protection against *M. tuberculosis* infection than L91 and BCG vaccines. It can be seen from the above studies that peptide-based vaccines using both HTL and CTL peptides as well as agonists or helper peptides can induce stronger CD4^+^ and CD8^+^ T cell immunity to improve their protective effect (115, 270).

Furthermore, two additional studies were performed to improve the immunogenicity and protection of Acr _91-110_ peptide by adding other peptides, such as TB10.4 _1-13_ (MSQIMYNYPAMLG), TB10.4 _78-94_ (ANTMAMMARDTAEAAKW), Rv0476 _1-19_ (MLVLLVAVL VTAVYAFVHA), CFP10 (Rv3874) _71-90_ (EISTNIRQAGVQ YSRADEEQ), Acr _21-40_ (LFAAFPSFAGLRPTFDTRLM), Hsp65 (Rv0440) _3–13_ (KTIAYDEEARR), Ag85B (Rv1886c) _56–64_ (PSMGRDIKV), 19 kDa (Rv3763) _51–61_ (KVVIDGKDQNV), Acr _31–50_ (LRPTFDTRLMRLEDEMKEGR) and Rv1733c _63–77_ (AGTAVQDSRSHVYAH) (202, 203). As expected, the results revealed that peptide-based vaccine with multi-peptides could induce significantly higher levels of IgG antibodies, IFN-γ, TNF, and IL-2 cytokines, and lower CFUs in lungs of C57BL/6 mice (211) or HLA-DR3 transgenic mice (202).

#### 3.2.4 Peptide-Based Vaccines Derived From PE/PPE Family Proteins

In *M. tuberculosis*, PE/PPE family has up to 167 members, most of which are located on the surface of *M. tuberculosis* or secreted out of the bacteria and can be well recognized by the host immune system. Despite the function of most PE/PPE family members is still unknown, accumulating studies indicate that PE/PPE family members related to the ESAT6 family are considered as “immunogenicity islands” due to their high immunogenicity and immunopathogenic (271; 248). This evidence suggests that PE/PPE proteins may be promising candidates for the design of peptide-based vaccines. Wu M et al. designed a multi-epitope DNA vaccine termed as pPES by grafting four Th1 epitopes ESAT-6 _1–20_ (MTEQQWNFAGIEAAASAIQG), Ag85B _241–255_ (VANNTRL WVYCGNGT), PE19 (Rv1791) _4–18_ (VTTQPEALAAAAANL), PPE25 (Rv1787) _241–255_ (AQFFASIAQQLTFGP), and a CTL epitope MTB10.4 (Rv0288) _3–11_ (QIMYNYPAM) into Hsp65 (Rv0440) scaffold protein (243 **).** pPES vaccination generated HSP65-specific antibodies, induced higher levels of IFN-γ^+^CD4^+^ T cell response, multi-functional CD4^+^ T cell response, cytotoxic CD8^+^ T cell response, and lower bacterial loads in lungs and spleens of mice (243). These data indicated that epitope grafting did not reduce the immunogenicity of HSP65 protein, and epitope grafting strategy may be a potential method to construct peptide-based vaccines. A study identified 21 immunodominant peptides from 167 proteins of the PE/PPE family and constructed three peptide-based vaccines by fusing these peptides to ESAT-6 protein, including PE peptides + ESAT-6 (shorten as PE-ESAT-6), PPE peptides + ESAT-6 (PPE-ESAT-6), and PE + PPE peptides + ESAT-6 (PE/PPE-ESAT-6) (244). The results showed that, compared to control, PE/PPE-ESAT-6 immunization induced significantly higher levels of IFN-γ, multifunctional CD4^+^CD44^+^CD62L^-^ T cells, and lower CFUs loads in lungs and/or spleens of C57BL/6J mice.

We recently developed a novel peptide-based vaccine termed as MP3RT (2). This vaccine is made up of six immunogenicity HTL peptides, including Mtb8.4 (Rv1174c) _69-83_ (LRNFLAAPPPQRAAM), PPE18 _115-129_ (RAELMILIATNLLGQ), PPE18 (Rv1196) _149-163_ (AAAMFGYAAATATAT), PPE68 (Rv3873) _138-152_ (DYFIRMWNQAALAME), RpfA (Rv0867c) _377-391_ (AYTKKLWQAIRAQDV), and TB10.4 (Rv0288) _21-35_ (YAGTLQSLGAEIAVE). To evaluate the effect of epitope MHC restriction on the immunogenicity and protective efficiency of the MP3RT vaccine, humanized C57BL/6 mice and wild- C57BL/6 mice were used. Our results showed that MP3RT induced significantly higher levels of IFN-γ and CD3^+^IFN-γ^+^ T lymphocytes and lower CFUs in the lungs and spleens of humanized mice rather than wild-type mice (2). The same method was used to evaluate other peptide-based vaccine named as ACP that only contains three peptides Ag85B (Rv1886c) _12-26_ (GRRLMIGTAAAVVLP), CFP21 (Rv1984c) _12-26_ (VVVATT LALVSAPAG), and PPE18 (Rv1196) _149-163_ (AAAMFGYAAATA TAT) (9). We found that although ACP induced significant humoral and cellular immune responses in humanized mice, its protective efficiency was not significantly better than that of the phosphate buffer solution (PBS) control. Taken together, these data once again demonstrated that grafting or fusion of multiple immunodominant epitopes on the protective antigen skeleton could significantly improve the immunogenicity and protection efficiency of the antigen, and these findings provide new ideas for the construction of peptide-based vaccines for TB.

#### 3.2.5 Peptide-Based Vaccines Derived From MPT64 (Rv1980c) Protein

The MPT64 protein is an immunogenic protein initially isolated from the culture filtrate of the BCG Tokyo strain (272). Previous studies have shown that MPT64 protein contains T or B cell epitopes, inducing strong humoral or cellular immune responses (273). Therefore, MPT64 protein is a promising candidate for constructing a peptide-based vaccine. Peptide MPT64 _190-198_ (FAVTNDGVI) has received more attention in recent years. Professor Zhu BD et al. developed a peptide-based vaccine Ag85B-MPT64(190-198)-Mtb8.4 (named as AMM). They investigated its immunogenicity and capacity to boost BCG-primed immunity in a DDA-BCG PSN adjuvant (dimethyl-dioctyldecyl ammonium bromide and BCG polysaccharide nucleic acid). They found that BCG-AMM prime-boost vaccination induced significantly higher levels of immune responses and better protection than BCG or AMM vaccination alone (245). Subsequently, they further confirmed this vaccine and developed a novel vaccine named AMH that consists of Ag85B-MPT64(190-198)- HspX (246). Compared with the mice receiving BCG only, the mice boosted with AMH, AMM, or their combination (AMH+AMM) showed significantly higher levels of specific antibodies and IFN-γ^+^ T cells. In addition, the mice boosted with the combination of AMM and AMH had substantially lower bacterial counts in the lungs, whereas mice boosted with AMH or AMM did not. Heterogeneity of protective effect of AMM vaccine in both studies may be related to vaccine dose. Analysis of the two studies showed that all parameters were identical except the vaccine dose. In their first study, they used 20 µg of the AMM vaccine, but in the second study, the vaccine dose was reduced to 10 µg. It indicates that vaccine dose significantly affects its protection efficiency, suggesting that future studies should select an appropriate vaccine dose to immunize mice to avoid vaccine failure due to this factor.

More recently, Zhu’s team modified and upgraded the AMM vaccine and constructed a new peptide-based vaccine called LT70, which consists of ESAT6-Ag85B-MPT64(190-198)-Mtb8.4-Rv2626c (247). They observed that LT70 was well recognized by T cells obtained from TB patients and LTBI volunteers and induced dramatically higher levels of cellular and humoral immunity as well as protective efficacy compared to BCG vaccine or PBS control in C57BL/6 mice. There were significant differences in experimental design between this study and the previous two studies. For example, the vaccine dose was adjusted to 13 μg, BCG-PSN adjuvant was replaced by Poly (I:C) adjuvant, and the route of the challenge was changed from intravenous injection to respiratory aerosol inhalation. These optimizations and improvements have contributed to the improved immune protection efficiency of the LT70 vaccine.

#### 3.2.6 Peptide-Based Vaccines Derived From Rv1733c Protein

Rv1733c is a probably conserved transmembrane protein of *M. tuberculosis* and belongs to dormancy survival regulon antigens (DosRs) related to LTBI (5). Rv1733c protein has been considered an immunopotent T cell candidate of the 45 top-ranking antigens (274). Black GF et al. compared the immunogenicity of 51 DosR regulon-encoded *M. tuberculosis* recombinant proteins among 131 individuals from Uganda, Gambia, and South Africa. They found that of the 51 DosRs, Rv1733c is one of the most frequently recognized DosRs in all three population groups (275). Furthermore, it has been shown that Rv1733c also induces strong IFN-γ response in T cells collected from tuberculin skin test positive (TST^+^) individuals (276) and a Rv1733c DNA prime followed by boosting with Rv1733c protein increased T cell proliferation and IFN-γ secretion in mice (277).

Thus, it can be seen that Rv1733c has good immunogenicity and is expected to be a new vaccine candidate for fighting against LTBI. Coppola M et al. investigated an SLP Rv1733c _57–84_ (IPFAAAAGTAVQDSRSHVYAHQAQTRHP) derived from Rv1733c protein and assessed its immunogenicity and protective capacity in HLA-DRB1*0301/DRA transgenic mice (203). After three times’ immunization, the mice vaccinated with Rv1733c SLP and TLR9 agonist CpG ODN1826 showed significantly higher levels of IFN-γ^+^ TNF^+^ and IFN-γ^+^ CD4^+^ T cells and Rv1733c protein-specific antibodies. Interestingly, compared with mice vaccinated with BCG only, the mice primed with BCG and boosted with Rv1733c SLP revealed the highest reduction in CFUs burdens in lungs (203). Furthermore, Geluk A et al. also evaluated the immunogenicity of another peptide Rv1733c _63–77_ (AGTAVQDSRSHVYAH) in HLA-DRB1*03:01/DRA transgenic mice. It was found that Rv1733c _63–77_ stimulated significantly higher levels of IFN-γ in splenocytes harvested from HLA-DR3 mice infected with *M. tuberculosis* and showed higher levels of IFN-γ^+^, TNF^+^, or IL-2^+^ CD4^+^ T cells (202). These data suggest that Rv1733c SLP may be a potential booster vaccine for TB.

## 4 Protein-Based Bacterial Vaccine Delivery System

In recent years, subunit vaccines, especially peptide-based vaccines with more single and safer components, have gradually become new vaccine forms. However, their weak immunogenicity makes it difficult to induce an adequate immune response and thus often need adding additional adjuvants. With the development of immunology, the delivery system, which aims to enhance antigens targeting secondary lymphoid organs and the activation of APCs, is continuously developed and applied in the vaccine design. Although many potential delivery systems have been widely explored in therapeutic vaccines, there have not been thoroughly studied in prophylactic vaccines because of the higher requirements for the safety of the materials usually applied in healthy people, even the elderly and children. With that in mind, more compatible and safer protein-based delivery vectors have great potential in prophylactic vaccine research (**Table 4**).

**Table 4:** Protein-based bacterial vaccine delivery systems.

Delivery systems	Antigens	Targeted pathogens	Adjuvant	References
**Self-assembled proteinaceous nanoparticles**				
VLP				
HBsAg	Capsular polysaccharide 33F	*S. pneumoniae*	—	(278)
	PRP polysaccharide	*H. influenzae type b*	—	(279)
	Vi polysaccharide	*S. typhi*	—	(279)
Qβ	TS3 and TS14 (capsular polysaccharides repeated units)	*S. pneumoniae*	—	(280)
AP205	O-polysaccharide	Shigella	—	(281)
Flock house virus	PA	*B. anthracis*	—	(282)
T4 Bacteriophage	PA	*B. anthracis*	—	(283)
	Mutated capsular antigen F1 and low-calcium-response V antigen	*Y. pestis*	—	(282)
Designable self-assembled nanoparticle				
	O-polysaccharide	Shigella	—	(284)
	O-polysaccharide	*S. Paratyphi A*	—	(284)
	O-polysaccharide	*K. pneumoniae*	—	(285)
**Live viral vector**				
	PAD4; PA	*B. anthracis*	—	(286–288)
	Cu-Zn SOD; IF3; L7/L12; Omp16; Omp19	Brucella	—	(289–292)
	D2 domain of FnbpB	*S. aureus*	—	(293)
**Bacterial vectors**				
Probiotics	PsaA; PspA; PspA5; PppA; PspC	*S. pneumoniae*	—	(294–300)
	Fusion of ST and LTB; F41; K99 fimbriae; β-Intimin fragment; Fusion of K99, K88 fimbriae; FaeG; FaeG with DC-targeting peptide; EspA and the Tir central domain; PapG	*E. coli*	Without, or LTB mutated LTA and LTB	(301–311)
	CTB	*V. cholerae*	—	(312)
	FliC or fusion of FilC and SipC	*S. enterica serovar Enteritidis*	—	(313, 314)
	PA; PA with DC-targeting peptide	*B. anthracis*	—	(315–319)
	L7/L12, Cu-Zn SOD, Omp31	Brucella	—	(320–322)
	ClfA and FnbpA; B-cell epitope, D3(22–33), from FnbpA	*S. aureus*	Without or Freund’s adjuvant	(323, 324)
	Hp0410; Urease B subunit	*H. pylori*	—	(325, 326)
	LcrV	*Y. pestis*	—	(327)
GEM	PppA; IgA1p; PpmA; SlrA	*S. pneumoniae*	—	(299, 328)
	PTd, FHA, and PRN	*B. pertussis*	—	(329)
	CUE	*H. pylori*	—	(330, 331)
	Omp31	Brucella	—	(322)
*S. enterica*	PA, PAD1 and 4, and PAD4	*B. anthracis*	—	(332)
Attenuated *S. typhimurium*	L7/L12; Fusion of L7/L12 and BLS	Brucella	—	(333, 334)
	SaEsxA and SaEsxB	*S. aureus*	—	(335)
*Ochrobactrum anthropi*	Cu-Zn SOD	Brucella	CpG	(336)
**OMV**				
	OMV components	*K. pneumoniae*	—	(337)
	OMV components	*B. pertussis*	—	(338–340)
	OMV components	*E. coli*	—	(341)
	OMV components	Shigella	Without or Alhydrogel	(342–345)
	OMV components	*V. cholerae*	—	(346, 347)
	OMV components	*S. typhimurium*	—	(348)
	OMV components	*S. typhi and paratyphi A*	—	(349)
	HlaH35L, LukE and extracellular vesicle (EV) components	*S. aureus*	—	(350, 351)
	OMV components	*H. pylori*	—	(352)
	OMV components	*Y. pestis*	—	(353)
Modified OMV				
Deposited onto bovine serum albumin nanoparticles	OMV components	*K. pneumoniae*	—	(354)
Lipid A-meditation	OMV components	*B. pertussis*	—	(355)
OMVs+Chitosan+Eudragit L-100	OMV components	*E. coli*	—	(356)
Encapsulated in polyanhydride nanoparticles	OMV components	Shigella	—	(357, 358)
Encapsulated in chitosan-tripolyphosphate particles and Eudragit L-100	OMV components	Shigella	—	(359)
Encapsulated in indocyanine green (ICG)-loaded magnetic mesoporous silica nanoparticles	EVs components	*S. aureus*	—	(360)
E. coli OMV	Glycan antigens (Polysialic acid (PSA) and T antigen)	Neisseria meningitidis group B	—	(361)
	HlaH35L, SpAKKAA, FhuD2, Csa1A, and LukE; SAcoagulase	*S. aureus*	—	(362, 363)

### 4.1 Self-Assembled Proteinaceous Nanoparticles

Some proteins can assemble into particles of a specific size under natural conditions and have been developed as great delivery systems. One kind is virus-like particles (VLPs) which are artificial nanostructures that self-assembled after the expression of viral capsid protein. It has been reported that poly (ribosylribitolphosphate) (PRP) polysaccharide of Hib was connected to Hepatitis B virus surface antigen (HBsAg) VLP *via* an adipic acid dihydrazide (ADH) spacer, and stronger IgG antibodies to both the PRP were induced than a commercial conjugate vaccine in mice (279). Similar, VLPs (e.g., Qβ and HBsAg) could also chemically load S. pneumonia capsular polysaccharide. The VLP vaccines could induce serotype-specific IgG antibodies. With synthetic biology and protein glycosylation system development, a new and simpler coupling method between polysaccharide antigens and protein has been explored. Li X. et al. successfully prepared the Shigella conjugate vaccine by using bacterial *in vivo* protein glycosylation reaction to couple the complete pathogenic bacterial polysaccharide to VLP (AP205) for the first time (281, 364). This VLP based conjugate vaccine showed better immune and protective effects in mice than the conventional vector. Besides, flock house virus VLP and bacteriophage T4 nanoparticle vaccine were explored and exhibited good protection against the challenge (282, 283). Designable self-assembled nanoparticle is another kind of self-assembled proteinaceous delivery vector used in pathogenic bacterial vaccines. Due to its modular design, it is a promising protein vector, which has shown good development potential in the development of vaccines such as viruses, but it seems to have just begun in the field of bacteria. Pan et al. developed a Nano-B5 system to produce self-assembled nano vaccines by fusion expression of bacterial B5 toxin and trimeric peptide and connected polysaccharide antigen through glycosylation in the pathogenic host (284). This particle was about 25 nm, which prolonged retention in draining lymph nodes and could stimulate strong cellular and humoral immune responses. Further, the system could be introduced into a modified *E. coli* host to prepare exogenous pathogenic bacteria, such as *Klebsiella pneumoniae*, nano-scaled conjugate vaccine and protect mice from systemic and pulmonary infection (285). Polysaccharide conjugate vaccine is considered the most successful bacterial vaccine at present. Although immunogenicity of carbohydrate antigen itself is very weak, it could be significantly improved by conjugating them (either synthetic short sugar chain or natural polysaccharide) with proteinaceous nanoparticles. Thus, self-assembled proteinaceous has excellent potential to enhance weak antigen immunogenicity and be used in the bacterial vaccine.

### 4.2 Viral Vector

Vaccines consist of a non-replicating virus that contains certain genetic material from the pathogen that needs to be immunized. It seems to be an ideal vaccine delivery system because of its natural viral structure, which can be well recognized by the immune system (365). The adenovirus vector is widely used (366) to develop a bacterial vaccine. Other viral vectors (e.g. influenza viral and semliki forest virus) have also been explored. McConnell M J et al. described a replication-incompetent adenovirus expressing domain 4 (D4) of *B. anthracis* protective antigen (PA) (Ad.D4), which could induce a more robust humoral and cellular immune responses than anthrax vaccine absorbed (AVA) (the only one FDA-approved anthrax vaccine which needs to be vaccinated six times within 18 months and enhanced once each year) (367) and provide complete protection against lethal spore challenge in single immunized mice (286). However, pre-existing immunity to Ad in humans may inhibit subsequent immunization-induced antibody responses. Influenza viral vectors may be another promising one for human use because of the lack of pre-existing immunity, safety and immunogenicity, which have been demonstrated in various models (chickens, ferrets and rhesus macaques and humans) (368–370). Tabynov K et al. developed recombinant influenza A viruses of the subtypes Н5N1 and H1N1 expressing *Brucella* protective antigen (ribosomal protein L7/L12 or Omp16) and strong cellular immune response and protection effect were induced (291). Moreover, these vaccines with adjuvant could provide long term protection for cattle and induced good cross-protection against *B. melitensis* infection in pregnant heifers and even sheep and goats (371–375). However, influenza viruses expressing *B. anthracis* PA unable to induced *in vitro* anthrax toxin neutralization activity antibodies, although the titers against PA were high (287, 376). Interesting, this situation could be solved by heterologous prime/boost immunization strategy, which may be attributed to the B-cell affinity maturation and Ig gene high-frequency mutation in germinal centres by combining different heterologous vectors. Moreover, antigen epitopes (PA_232–247_ and PA_628–637_) also could be expressed in a plant-virus Tobacco Mosaic Virus (TMV). However, immunized mice showed almost no protection (377). Possible reasons may be the too weak immunogenicity of short epitopes or plant virus self, and a prime/boost immunization strategy can be tried.

### 4.3 Lactic Acid Bacteria Vector

Lactic acid bacteria are commonly used as delivery vehicles because they are safe and human friendliness and can stimulate mucosal and systemic immune responses through mucosal pathways. Many kinds of lactic acid bacteria (e.g. *L. reuteri*, *L. casei*, *L. plantarum* and *L. plantarum*) have been used as delivery vectors, of which *L. casei* is the most studied (378). Lactic acid bacteria are generally applied for the vaccine design against intestinal and respiratory infection, and there are main two strategies for antigen delivery. One is to express antigen *in vivo* directly. Studies have shown that an oral vaccine expressing *E.coli* F41 in *L. casei* can stimulate strong systemic and local mucosal immune responses simultaneously and protect mice from the lethal challenge, even still achieving more than 80% protection nine weeks after the last immunization (303, 304). In addition, Yu M et al. found that when the antigen was co-expressed with B5 toxins (such as LTB), it induced a more robust mucosal immune response and provided 100% protection (311). Moreover, even some capsular polysaccharides, such as type 3 and type 14 of *S. pneumonia*, have been successfully expressed in *L. lactis*, and the immune response of type 3 vaccine was detected, showing that *L. lactis* is a potential host for capsule vaccine antigens (379). Another strategy is using a non-genetically modified gram-positive enhancer matrix (GEM) particle for antigen delivery. The particles were prepared from living bacteria and had no nucleic acid and cytoplasmic components while maintaining the size and cell wall components of the bacteria. Multiple *S. pneumonia* protein antigens (e.g., PppA, PpmA, SlrA and IgA1p) have been anchored on the particles by a lactococcal peptidoglycan binding domain and shown to be efficacious against pneumococci in animal models (299, 328, 380). In addition, GEM loading epitope antigens also showed a significant effect. For example, when the *H. pylori* multi-epitope vaccine (CUE) (based on CTB fusing with T and B cell epitopes from *H. pylori* urease A and B subunits) was displayed on the surface of GEM, these prophylactic and therapeutic effects in orally immunized mice could further enhanced by inducing mucosal specific antibody responses and local Th1/Th17 cell-mediated immune response (330, 331), which was an optimal immunity type agains*t H. pylori* infection (381, 382).

### 4.4 Outer Membrane Vesicles (OMVs)

A large number of gram-negative bacteria naturally could produce extracellular OMVs, which is from 50 to 250 nm in diameter, suitable for targeting and being phagocytized by APCs (383). OMV contains many components, such as outer membrane proteins (OMPs) and lipoproteins, which are conducive to immune response and various immunogenic antigens. At present, the use of OMVs has become a very promising vaccination strategy. OMVs from many pathogenic bacteria (e.g. Klebsiella pneumoniae, *B. pertussis, E. coli, Shigella, Vibrio cholerae, Salmonella*, *Helicobacter pylori* and *Neisseria meningitidi*s group B) has been proved to have the ability to stimulate humoral and cellular immune responses and provide good protective effect after immunization (337, 384–386).

Because natural OMVs often contains toxic components like LPS, which could induce host inflammatory responses, many studies have focused on reducing the toxicity of OMVs by deleting the lipid A related genes (e.g. *msbB, htrB*, *pagP, lpxL*, or pagL) (355, 387–390) or toxin genes (391, 392). For example, Kim S H et al. generated OMVs from *E. coli* O157:H7 with the mutation of *msbB* (encoding an acyltransferase catalyzing the final myristoylation step during lipid A biosynthesis) and Shiga toxin A StxA. The reduced toxicity OMVs were immunized by eyedrop in BALB/c mice and showed that it was safe and could induce both humoral and mucosal immune (tear, saliva, and fecal) responses, which is enough to protect the vaccinated animal from the challenge of the lethal HUS-causative agent (wtOMVs) (341). Another example was that the knocking out of *lpxL*, which is involved in lipid A biosynthesis, in *N. meningitidis* could result in at least a 200-fold decrease in pyrogenicity than wild-type OMV. The protective effect can be largely restored by adding adjuvants used in humans (393). In addition, Sinha R et al. reported that the OMV-mediated toxicity could be significantly reduced by being pre-treated with all-trans retinoic acid (ATRA), active metabolites of vitamin A, which are anti-inflammatory and mucosal adjuvant properties, and the immunity was enhanced (394). Fredriksen J H et al. produced a group B *N. meningitidis* OMV vaccines by including an additional step of detergent extraction (395). The detergent extracted outer membrane vesicles contain much fewer LPS (5–8%) and have been helpful in several countries (396). Moreover, a hydroquinone non-pathogenic OMV from *E. coli* was developed as delivery vehicles by expressing group B glycan antigens (361). However, most bacterial capsular polysaccharides in gram-positive bacteria are difficult to express in gram-negative bacteria *E. coli* efficiently. The difference of membrane structure and the polysaccharide gene cluster is relatively large (mostly more than 10 kbp), making it difficult for cloning.

OMVs were usually combined with other delivery systems to meet some specific requirements. For example, to solve the problems of the poor size uniformity and low stability of OMV, Wu, G. et al. produced a 70-90 nm sized OMV (from *K. pneumoniae*) based nanovaccine by depositing the hollow-structured OMVs onto bovine serum albumin nanoparticles. As a result, the OMV could be reinforced from the core-shell structure. The protecting effect against carbapenem-resistant K. pneumoniae (CRKP) was significantly improved after vaccination (354). Camacho A I et al. found that when *Shigella* OMVs were encapsulated in polyanhydride nanoparticles, a stronger Th1 immune response, which was more needed against intracellular bacteria, was induced (357, 397). OMVs also be encapsulated in biopolymer chitosan, which was used to prepare nanogel particles by ionotropic gelation with tripolyphosphate. After being coated with an enteric polymer, mice were administrated orally and showed better protection against infection after 78 days of immunization, whereas free OMVs have no protection (359). Therefore, although OMV as a delivery carrier faces some problems, it can achieve the expected effect through further optimization and transformation.

### 4.5 Liposome

The liposome is a kind of phospholipid bilayer sphere formed *via* self-assembly in water and proved to be a safe and effective delivery system. Although liposomes do not belong to protein delivery carriers, we will still review their application in vaccine design, especially for peptide-based vaccines, because of their wide use (398). The versatility and plasticity characteristics of liposomes make them designable according to different parameter requirements, such as lipid composition, charge, size, entrapment and location (399). Besides loading various antigens in a liposome, adjuvants and/or functional molecules could also be loaded quickly to enhance further the immune effect (400). For peptide antigens, they were usually coupled to liposomes *via* lipid core peptide technology, which consists of an oligomeric polylysine core conjugated to a series of lipoamino acids for anchoring of the antigen (401, 402), and this strategy has been frequently utilized in Group A Streptococcal (GAS) vaccine studies (403–405). Ghaffar et al. developed a cationic liposome through the film hydration method with dimethyldioctadecylammonium bromide (DDAB). Lipopeptides antigens, entrapped by the liposome, could induce both mucosal and systemic response for a long time in intranasally immunized mice (403). In addition, the high-level antibody reaction was further confirmed in various sizes (70 nm to 1000 nm) of the carriers (406). In addition, some polymer, such as polyethylenimine (PEI), which could attach to the cells’ surface and deliver cargo into endosomal and cytosolic compartments, was introduced in the lipopeptide-based vaccine design. PEI incorporated in liposome peptide vaccine could induce significant specific mucosal and systemic antibodies, which effectively opsonize multiple isolates of clinically isolated GAS (407). Further, they found that the ratio of PEI, rather than molecular weight, present in the liposome vaccines impact immune response (408). Besides, the addition of some functional elements can realize the immune enhancement of vaccines. For example, Yang et al. designed a cell-penetrating peptides (CPPs)-liposome delivery system on the liposomal nanoparticles, in which CPP could enhance both cellular and humoral immune responses through direct delivery of antigen into the cytoplasm and from the endocytic pathway (409–411). Nasal immunization of the vaccine in mice could induce antibodies that showed high opsonic activity against clinically isolated GAS strains (412). Liposomes have also been used in the study of TB vaccines. Dimethyldioctadecylammonium (DDA) could self-assemble into closed vesicular bilayers in water similar to liposomes and was known as an effective adjuvant for eliciting cellular and humoral responses (413, 414). However, the physical instability of the DDA liposomes limits its application. To solve this problem, Davidsen et al. incorporated a glycolipid trehalose 6,6′-dibehenate (TDB), comprising a 6,6′-diester of α,α′-trehalose with two long 22-carbon acyl chains, into the DDA liposome bilayers. By loading tuberculosis vaccine antigen Ag85B–ESAT-6 fusion protein and immunization of mice, a robust specific Th1 type immune response was induced (415). The post-challenge bacterial growth of *M. bovis* BCG was reduced in adult or neonatal murine (416), suggesting the increase of adjuvant efficacy of DDA liposomes. In conclusion, the characteristics of liposomes endow them with more designability, and some deficiencies can be solved by further transformation and optimization so that liposomes have great potential in peptide vaccine delivery design.

## 5 Animal Models for Peptide-Based Vaccines

Many animal models have been utilized to develop TB vaccines, ranging from expensive none-human primates (NHPs) to small non-mammals such as zebrafish. In comparison, NHP, which can well reflect the human immune response and susceptibility to TB, has been used in many preclinical experiments. At the same time, smaller animal models such as mice, rats, guinea pigs, rabbits and zebrafish are generally more suitable for studying narrower aspects of the immune response to Mycobacterium tuberculosis, such as granuloma formation, susceptibility to different strains, or immunogenicity of vaccine candidates.

NHP represents one of the most frequently used and most important models when testing vaccines against *M. tuberculosis* infection. This model has significant similarities in human physiology, genome, and immune response (417, 418). Rhesus macaques and cynomolgus are the most commonly used NHPs in TB infections for vaccine evaluation (417, 419–421). Today, the NHP models have become indispensable for the preclinical evaluation of vaccine effects. However, some of the difficulties in using NHP models to evaluate anti-tuberculosis vaccines are the considerable investment requirements for the maintenance and use of BLS-3 biosafety facilities, the lack of commercial molecular and immune reagents, and the timely availability of sufficient animals. Compared with the NHP model, the advantages of using the mouse model include the availability of genetically modified strains, more common molecular and immunological reagents on the market, and lower cost of feeding and specialized containment facilities. These characteristics ensure that the mouse model is always the first choice for TB vaccine research, especially for exploratory studies on vaccines before preclinical evaluation using NHP models. Although common mouse strains exampled as C57BL/6 and BALB/c are often not susceptible to tuberculosis infection, they are still widely used in vaccine research. To date, most *M. tuberculosis* peptides are discovered in the C57BL/6 model (**Table 3**).

The use of mice is ubiquitous in scientific research. Still, the experimental results obtained on rodents, and primarily murine, in reality, are often very different from the clinical immune response of humans. Moreover, this difference in immune response has a certain relationship with the species differences between animals and humans, causing the development of many novel vaccines and drugs to stagnate or even fail to continue when they reach clinical trial phase I/II. Therefore, the development of small animal models that can more accurately reflect the characteristics of human immune response is a problem that deserves special attention.

MHC is one of the gene groups with the most polymorphism in humans and mammals. It induces and regulates innate immunity and adaptive immune response and participates in the development and maturation of T lymphocytes, the presentation of exogenous/endogenous antigens and immune signals, as well as the establishment of central immune tolerance (422). At the same time, MHC is closely related to the occurrence and progression of many autoimmune diseases and chronic diseases and has essential biological functions and significance. For example, the HLA-A11 subtype is closely associated with the occurrence of many infectious diseases, such as familial otosclerosis (423, 424), TB (425), leprosy (426), epilepsy and cytomegalovirus infection (427), etc. In addition, increased specific expression of the HLA-A11 gene was found in patients with upper laryngeal cancer (428) and osteosarcoma (429). Furthermore, HLA-A11, DR3 and DR4 subtypes played a synergistic role in the occurrence of autoimmune hepatitis (430). In addition to HLA-A11, other individual MHC subtypes also play a significant role in the disease process after HIV-1 infection (431–435). In recent years, how to use and exert the biological functions of HLA through animal models has gradually become a research hotspot in animal models, and the first is the development of new vaccines based on MHC-restricted CTL and HTL epitopes.

Mice and humans share more than 95% of genes and about 80% of genetic material. As small animal models, mice are widely used in vaccines and drugs preclinical trials (436). Although the MHC of the mouse (H-2) and human (HLA) are very similar in structure and function, there are still significant differences in the presentation of antigens (437), and the dominant antigen peptides presented also have different structural characteristics. Therefore, the MHC humanized mouse has become an essential model for epitope research and the development and evaluation of epitope vaccines.

In recent years, MHC humanized mouse models have played a vital role in developing and evaluating disease immune pathogenic mechanisms, vaccines, and drugs. This mouse model has also undergone a continuous development and progress stage. In the first stage, the whole HLA molecule is usually directly transferred into the mouse genome, such as HLA-B27 mice (438), HLA-B7 mice (439), HLA-A2 mice (440), HLA-Cw3 mice (441) and other early developed models. However, the binding force between the α3 functional region in the human HLA molecule and the mouse CD8 is weak (442–444). At the same time, the presence of mouse MHC (H-2) leads to competitive inhibition of mouse H-2-I restricted immune response to human HLA-I restricted immune response (445). In this stage, the mouse immune response is still dominant in the model, which cannot reflect the function and role of human HLA molecules alone in the immune response. Studies have shown that when H-2 is present in mice, the expression of HLA transgenic molecules on the surface of mouse lymphocytes is significantly reduced (446). In the second stage, the scientists improved the MHC humanized mouse model through two methods. One is to transfer the gene fragment encoding human CD4^+^ or CD8^+^ into the mouse genome so that the mouse can express both murine and human CD4^+^ or CD8^+^ molecules (447), which can effectively improve the binding efficiency of HLA and CD4 or CD8 accessory molecules, and efficiently start the second signal of antigen presentation. Another method is to optimize the structure of HLA transgenic molecules, replace the transmembrane α3 functional region with murine α3 functional region, and construct a chimeric HLA molecule (HHM). The murine α3 structure can promote HLA and mouse CD4 or mouse CD8. Representative mouse models include the HLA-B27 (HHM) mice model (448, 449). After optimization of the above two methods, the mouse model can produce a certain HLA-restriction-specific response. However, most cellular immune response is still regulated by mouse H-2 molecules. In the third stage, the researchers adopted a new construction strategy to design transgenic vectors and constructed a chimeric transgenic vector (HHD) (450) of HLA and H-2, including the promoter of HLA-I, light chain β2m, and heavy chain. The α1 and α2 functional regions, the α3 functional region and the transmembrane region of H-2-Db, and the important components β2m and IAβ of mouse H-2 are also knocked out. For example, in the HLA-A11 humanized mouse model (451), the α3 functional area of ​​mouse H-2 is used to replace the α3 functional area of ​​human HLA-A11, which effectively enhances the binding force between the human HLA-A11 molecule and the mouse CD8 and avoids the complexity of transferring human CD8 into the mouse genome. At the same time, knock out the important components β2m and IAβ (the β chain of the IA molecule) of the mouse-derived H-2 I and II molecules, and replace them with fragments larger than the originals. Such pseudogene fragments of the gene achieving the purpose of silencing the expression of mouse H-2 molecules. Therefore, in this type of HLA-I humanized mice, the competitive inhibition of the H-2-I restricted reaction is eliminated, and only the HLA-I restricted immune response can be produced. The humanized mouse model of HLA-II was also constructed using the same strategy. The resulting HLA-I/II humanized mouse model was replaced with human HLA molecules at the level of MHC-I and II molecules. HLA exerts a restrictive function in antigen presentation, regulates the immune response in mice, and enables MHC mice to be “humanized” to a greater extent, which can more effectively simulate human immune response at the HLA level. The representative HLA-A2/DR1 and HLA-A11/DR1 transgenic mouse models have been well applied in HIV、EBOV and SARS-CoV-2 epitope screenings (451–453). Encouragingly, the MHC humanized mouse model has been well used for TB peptide-based vaccine research (2, 9, 198, 202, 203).

Although the MHC humanized mouse model can effectively simulate the human immune restriction and affect the immune function. However, as a better model for epitope vaccine research and evaluation, MHC humanized mouse models have two problems that need to be optimized in future development. On the one hand, it makes MHC humanized mouse models sensitive to pathogens. On the other hand, mice are not susceptible to many human pathogens or cannot be infected due to differences in receptors. For example, regular mice are not sensitive to SARS-CoV-2, SARS-CoV, MERS-CoV, and many subtypes of influenza viruses. Therefore, it requires changing the virus (constructing a mouse-adapted strain) or changing the animal (pathogen-receptor humanized mouse) to obtain a sensitive mouse model. Another aspect is to combine MHC humanized mice with immunodeficient mice for better humanized immune reconstitution. At present, although NOD/SCID mice have played a critical role in the research fields of immune transplantation and tumor immunity, the transplanted human-derived cells have undergone development and differentiation in the mouse thymus. However, the MHC obtained restriction is still restricted by mouse H-2, and it is impossible to carry out research on specific HLA-restricted CTL and Th immune responses. Therefore, through the combination of MHC humanized mice and immunodeficient mice, immune reconstitution can be humanized, and a more real humanized immune system can be realized.

## 6 Conclusions and Future Perspectives

Vaccination has been considered as the most effective strategy to eliminate TB infection. Accumulating studies have showed that peptide-based vaccines are promising vaccine candidates for preventing and controlling TB due to their advantages, such as aggregation of immunodominant epitopes, preservation of peptide structure, good stability, easy to store and transport, lower cost, and decreased side effects. Furthermore, the rapid development of bioinformatics technology provides a tool for predicting and constructing peptide-based vaccines, which dramatically saves time and reduces the cost of peptide-based vaccine research. Herein, we give a detailed description of how to design a peptide vaccine using an immunoinformatics approach, including determination of protective antigens, T and B cell epitope prediction, screening of immunodominant epitopes, and selection of selection linkers, adjuvant or helper peptides, codon optimization, and in *silico* analysis. We further reviewed the peptide-based vaccine candidates worldwide based on this basic knowledge. We found that 150 previous articles related to peptide-based vaccines for TB are being investigated in pre-clinical studies, including 76 studies in epitope screening and prediction, 45 studies in evaluating immunogenicity, 8 studies in peptide-based vaccine construction, and 21 in assessing vaccine efficacy in animal models. However, some drawbacks of peptide-based vaccines should not be ignored, such as weak immunogenicity for a single peptide, MHC restriction, and high requirements for animal models.

In the future, these disadvantages can be solved by the following strategies: (1) a detailed understanding of the potential cellular and molecular mechanisms involved in peptide-based vaccine immunity is the key to improving its immunogenicity and protective efficiency (454); (2) improving the vaccine construction techniques, including broad antigen repertoire, SLPs, conjugation and palmitoylation of peptides, grafting epitopes into a protective antigen; (3) using appropriate linkers, helper peptides, TLR agonists, adjuvants, and potential delivery systems to enhance the immunogenicity; (4) primed with BCG and boosted with peptide-based vaccines; (5) employing transgenic animal models with human HLA molecules to evaluate peptide-based vaccines.

## Author Contributions

Conceptualization: XW and WG. Data curation: WG, CP, PC, JW, and GZ. Formal analysis: WG. Funding acquisition: WG. Methodology: WG, CP, PC, JW, and GZ. Software: WG. Writing - original draft: WG, CP, and GZ. Writing - review & editing: WG and XW. All authors contributed to the article and approved the submitted version.

## Funding

This study was funded by the National Natural Science Foundation of China (Grant No. 81801643), Beijing Municipal Science & Technology Commission (Grant No. 19L2152), and Chinese PLA General Hospital (Grant No. QNC19047).

## Conflict of Interest

The authors declare that the research was conducted in the absence of any commercial or financial relationships that could be construed as a potential conflict of interest.

## Publisher’s Note

All claims expressed in this article are solely those of the authors and do not necessarily represent those of their affiliated organizations, or those of the publisher, the editors and the reviewers. Any product that may be evaluated in this article, or claim that may be made by its manufacturer, is not guaranteed or endorsed by the publisher.

## Supplementary Material

Supplementary Table S1 Summary of studies related with peptide or epitope candidates against Mycobacterium tuberculosis.

## Abbreviations

ADH, adipic acid dihydrazide; ATRA, all-trans retinoic acid; ANN, Artificial neural network; APCs, antigen presenting cells; AUCs, areas under the curve; BCG, Bacillus Calmette-Guérin; BMDCs, bone marrow derived DCs; BMDMs, bone marrow-derived macrophages; CAI, Codon Adaptation Index; CFP-10, culture filtrate protein 10; CRKP, carbapenem-resistant K. pneumoniae; CTB, cholera toxin subunit B; CTL, cytotoxic T-lymphocytes; COVID-19, coronavirus disease 2019; DCs, dendritic cells; DosRs, dormancy survival regulon antigens; ELISpot, enzyme-linked immunospot; ESAT-6, early secreted antigenic target 6; GEM, gram-positive enhancer matrix; HBD, human β-defensin;, HBHA, heparin binding hemagglutinin; HBsAg, Hepatitis B virus surface antigen; HTL, helper T lymphocytes; HIV, human immunodeficiency virus; HLA, human leukocyte antigen; HMM, Hidden Markov Model; IEDB, Immune Epitope Database and Analysis Resource; IFN-γ, interferon-γ; IL, Interleukin; IP-10, interferon gamma inducible protein 10; LTBI, latent TB infection; MCC, Matthew’s correlation coefficient; MDR-TB, drug-resistant and multidrug-resistant TB; MM6, MonoMac6 human monocytes; MHC, major histocompatibility complex; NCBI, National Center of Biotechnology Information; NMR, nuclear magnetic resonance; NK, natural killer; ODN, oligonucleotides; OMVs, Outer membrane vesicles; Pam2Cys, dipalmitoyl-S-glyceryl cysteine; PA, protective antigen; PAMPs, pathogen associated molecular patterns; PBS, phosphate buffer solution; pDCs, plasmacytoid dendritic cells; PLS, partial least squares; pMHC, major histocompatibility complex presented antigenic peptides; PRP, ribosylribitolphosphate; PSMα4, phenol-soluble modulin α4; QSAR, Quantitative Structure Activity Relationship; RD, region of difference; Rpfs, resuscitation-promoting factors; RplL, 50S ribosomal protein L7/L12; SCXRD, single-crystal X-ray diffraction; SLPs, synthetic long peptides; SMM, Stabilized matrix method; SVM, Support Vector Machine; TB, tuberculosis; Th, helper T-lymphocytes; TLR, toll-like receptor; TMV, Tobacco Mosaic Virus; TNF-α, tumor necrosis factor-α; TST, tuberculin skin test; VLPs, virus-like particles; WHO, World Health Organization.

## Glossary

**Table:**

ADH	adipic acid dihydrazide
ATRA	all-trans retinoic acid
ANN	Artificial neural network
APCs	antigen presenting cells
AUCs	areas under the curve
BCG	Bacillus Calmette-Guérin
BMDCs	bone marrow derived DCs
BMDMs	bone marrow-derived macrophages
CAI	Codon Adaptation Index
CFP-10	culture filtrate protein 10
CRKP	carbapenem-resistant K. pneumoniae
CTB	cholera toxin subunit B
CTL	cytotoxic T-lymphocytes
COVID-19	coronavirus disease 2019
DCs	dendritic cells
DosRs	dormancy survival regulon antigens
ELISpot	enzyme-linked immunospot
ESAT-6	early secreted antigenic target 6
GEM	gram-positive enhancer matrix
HBD	human β-defensin;
HBHA	heparin binding hemagglutinin
HBsAg	Hepatitis B virus surface antigen
HTL	helper T lymphocytes
HIV	human immunodeficiency virus
HLA	human leukocyte antigen
HMM	Hidden Markov Model
IEDB	Immune Epitope Database and Analysis Resource
IFN-γ	interferon-γ
IL	Interleukin
IP-10	interferon gamma inducible protein 10
LTBI	latent TB infection
MCC	Matthew's correlation coefficient
MDR-TB	drug-resistant and multidrug-resistant TB
MM6	MonoMac6 human monocytes
MHC	major histocompatibility complex
NCBI	National Center of Biotechnology Information
NMR	nuclear magnetic resonance
NK	natural killer
ODN	oligonucleotides
OMVs	Outer membrane vesicles
Pam2Cys	dipalmitoyl-S-glyceryl cysteine
PA	protective antigen
PAMPs	pathogen associated molecular patterns
PBS	phosphate buffer solution
pDCs	plasmacytoid dendritic cells
PLS	partial least squares
pMHC	major histocompatibility complex presented antigenic peptides
PRP	ribosylribitolphosphate
PSMα4	phenol-soluble modulin α4
QSAR	Quantitative Structure Activity Relationship
RD	region of difference
Rpfs	resuscitation-promoting factors
RplL	50S ribosomal protein L7/L12
SCXRD	single-crystal X-ray diffraction
SLPs	synthetic long peptides
SMM	Stabilized matrix method
SVM	Support Vector Machine
TB	tuberculosis
Th	helper T-lymphocytes
TLR	toll-like receptor
TMV	Tobacco Mosaic Virus
TNF-α	tumor necrosis factor-α
TST	tuberculin skin test
VLPs	virus-like particles
WHO	World Health Organization
